# Characteristics of a Regulator of G-Protein Signaling (RGS) *rgsC* in *Aspergillus fumigatus*

**DOI:** 10.3389/fmicb.2017.02058

**Published:** 2017-10-23

**Authors:** Young Kim, In-Beom Heo, Jae-Hyuk Yu, Kwang-Soo Shin

**Affiliations:** ^1^Department of Biological Science, Daejeon University, Daejeon, South Korea; ^2^Departments of Bacteriology and Genetics, University of Wisconsin-Madison, Madison, WI, United States

**Keywords:** RGS, *Aspergillus fumigatus*, development, stress response, virulence, transcriptome

## Abstract

The regulator of G-protein signaling (RGS) proteins have a conserved RGS domain that facilitates the intrinsic GTPase activity of an activated Gα subunit of heterotrimeric G protein, thereby attenuating signal transduction. Among six predicted RGS proteins in the opportunistic human pathogenic fungus *Aspergillus fumigatus*, only three (FlbA, GprK, and Rax1) have been studied. The unexplored RgsC composed of the Phox-associated (PXA), RGS, Phox homology (PX), and Nexin_C superfamily domains is highly conserved in many ascomycete fungi, suggesting a crucial role of RgsC in fungal biology. To address this, we have investigated functions of the *rgsC* gene. The deletion (Δ) of *rgsC* causes impaired vegetative growth and asexual development coupled with reduced expression of key developmental regulators. Moreover, Δ*rgsC* results in accelerated and elevated conidial germination regardless of the presence or absence of an external carbon source. Furthermore, Δ*rgsC* causes reduced conidial tolerance to oxidative stress. In addition, activities and expression of catalases and superoxide dismutases (SODs) are severely decreased in the Δ*rgsC* mutant. The deletion of *rgsC* results in a slight reduction in conidial tolerance to cell wall damaging agents, yet significantly lowered mRNA levels of cell wall integrity/biogenesis transcription factors, indicating that RgsC may function in proper activation of cell wall stress response. The Δ*rgsC* mutant exhibits defective gliotoxin (GT) production and decreased virulence in the wax moth larvae, *Galleria mellonella*. Transcriptomic studies reveal that a majority of transporters is down-regulated by Δ*rgsC* and growth of the Δ*rgsC* mutant is reduced on inorganic and simple nitrogen medium, suggesting that RgsC may function in external nitrogen source sensing and/or transport. In summary, RgsC is necessary for proper growth, development, stress response, GT production, and external nutrients sensing.

## Introduction

Heterotrimeric G-protein (G-protein) signaling plays pivotal roles in sensing and responding to internal/external signals and various stresses. At upstream, a canonical G-protein signaling pathway is typically controlled by three components; G-protein coupled receptors (GPCRs), regulators of G-protein signaling (RGS), and heterotrimeric G proteins composed of α, β, and γ subunits (Lafon et al., 2005; Yu, 2006). RGS proteins harbor a conserved RGS domain that interacts with an activated Gα subunit and modulate the G-protein signaling pathways (Chidiac and Roy, 2003; McCudden et al., 2005). In filamentous fungi, RGS proteins play crucial roles in upstream regulation of vegetative growth, development, secondary metabolism, and virulence (Bayram and Braus, 2012).

In the human pathogenic fungus *Aspergillus fumigatus*, six genes predicted to encode RGS domain proteins have been identified (*flbA, gprK, rgsA, rax1, rgsC*, and *rgsD*). FlbA was shown to attenuate the GpaA (Gα)-dependent signaling pathway (Mah and Yu, 2006). It has been reported that the putative hybrid GPCR-RGS protein GprK also plays an important role in upstream regulation of G-protein signaling and contributes to proper asexual sporulation, gliotoxin (GT) production, and oxidative stress responses (Jung et al., 2016). Recently, Rax1 was shown to positively control vegetative growth and asexual development, and modulate trehalose amount and cell wall melanin levels in conidia, and conidia resistance against hydrogen peroxide (Igbalajobi et al., 2017).

RgsC is similar to *Saccharomyces cerevisiae* Mdm1 (McConnell et al., 1990), which is required for proper transmission of the nuclei and mitochondria from mother to daughter cells (Fisk and Yaffe, 1997). The Mdm1 protein confers a series of punctate structures distributed throughout the cytoplasm (McConnell and Yaffe, 1992). The *mdm1* null mutant fails to transmit mitochondria from the mother cell into the growing bud, simultaneously, suggesting that the Mdm1 protein network has a central function in facilitating organelle inheritance in the budding yeast (McConnell and Yaffe, 1992). However, Mdm1 does not harbor an RGS domain. The domain structure of RgsC of filamentous fungi is quite different from that of the budding yeast. RgsC of filamentous fungi contains the central RGS domain, the C-terminal PhoX homology (PX), and the N-terminal PhoX-associated (PXA) domain identified as a phosphoinositides (PI)-binding motif. The RgsC-type domain architecture has been found in more than 100 eukaryotic proteins with diverse functions (Ponting, 1996; Sato et al., 2001; Xu et al., 2001; Ellson et al., 2002). The PX domain might participate in protein trafficking and signal transduction by binding to PI (Sato et al., 2001). RGS-PX1 is known to play a bifunctional role as a GTPase-activating protein for Gαs and a sorting nexin protein (Zheng et al., 2001). While a potential role of RgsC in coordinating heterotrimeric G-protein signaling, hyphal extension, nuclear positioning, and vesicular trafficking has been speculated in filamentous fungi (Han et al., 2004b), no functional studies have been carried out.

In the present paper, we report the functional characterization of *rgsC* in *A*. *fumigatus*, and present a series of data elucidating the roles of RgsC in governing vegetative growth, asexual sporulation, germination, stress response, GT production, and virulence.

## Methods

### Strains and culture conditions

Glucose minimal medium (MMG) and MMG with 0.1% yeast extract (MMY) with appropriate supplements were used for general culture of *A*. *fumigatus* strains (Käfer, 1977). For pyrimidine and arginine auxotrophic mutant strain (AF293.6) (Xue et al., 2004), MMY was supplemented with 5 mM uridine, 10 mM uracil (for *pyrG1*), and 0.1% arginine (for *argB1*). For liquid submerged culture and phenotypic analyses on air-exposed culture were performed as described previously (Jung et al., 2016). To examine secondary metabolite production, spores of relevant strains were inoculated 50 ml of liquid MMY and incubated at 250 rpm at 37°C for 4 days.

### Generation of the *rgsC* deletion mutant

The oligonucleotides used in this study are listed in Supplementary Table 1. The *rgsC* gene was deleted in *A*. *fumigatus* AF293.6 (*pyrG1 argB1*) strain (Xue et al., 2004). The deletion construct generated employing double-joint PCR (DJ-PCR) (Yu et al., 2004) containing the *Aspergillus nidulans* selective marker (*AnargB*^+^) with the 5′ and 3′ franking regions of the *rgsC* gene was introduced into the recipient strain AF293.6 (Szewczyk et al., 2006). The selective marker was amplified from *A*. *nidulans* FGSC4 genomic DNA with the primer pair oligo 214/oligo 215. The *rgsC* null mutant was isolated and confirmed by PCR, followed by restriction enzyme digestion (Supplementary Figure 1; Yu et al., 2004). To complement *rgsC* null mutant, a single joint PCR (SJ-PCR) method was used (Yu et al., 2004). The ORF of *rgsC* gene with a promoter and terminator was amplified with primer pairs where the 3′ reverse primer carries overlapping sequences with the *ptrA* gene's 5′ end. Amplification of the *ptrA* gene was carried out with primer pairs where the 5′ forward primer carries overlapping sequences with *rgsC* gene's 3′ end. The final amplicon was amplified with the nested primer pair oligo 781/oligo 731 and introduced into a Δ*rgsC* strain.

### Nucleic acid isolation and manipulation

To isolate genomic DNA from *A*. *fumigatus*, about 10^6^ conidia were inoculated in 2 ml of liquid MMY, and stationary cultured at 37°C for 24 h. The mycelial mat was collected and squeeze-dried, and genomic DNA was isolated as described (Yu et al., 2004). The deletion mutant was confirmed by PCR amplification of the coding region of the gene followed by restriction enzyme digestion of the PCR amplicon. Total RNA isolation was carried out as previously described (Han et al., 2004a; Mah and Yu, 2006). Quantitative RT-PCR (qRT-PCR) assays were performed according to the manufacturer's instruction (Qiagen, USA) using a Rotor-Gene Q (Qiagen, USA). Each run was assayed in triplicate in a total volume of 20 μl containing the RNA template, One Step RT-PCR SYBR Mix (Doctor Protein, Korea), reverse transcriptase, and 10 pmole of each primer (Supplementary Table 1). Reverse transcription was performed at 42°C for 30 min. PCR conditions were 95°C/5 min for one cycle, followed by 95 and 55°C/30 s for 40 cycles. Amplification of one single specific target DNA was checked by melting curve analysis (+0.5°C ramping for 10 s, from 55 to 95°C). The expression ratios were normalized to EF1α expression and calculated according to the ΔΔCt method (Livak and Schmittgen, 2001).

### Phenotypic analyses

Germination rates were measured as previously described with a slight modification (Ni et al., 2005). To examine germination levels, conidia of WT and mutant were inoculated in 5 ml of liquid MMY, or liquid medium lacking a carbon source, and incubated at 37°C. Levels of germination were examined every 2 h after inoculation under a microscope. Various media were used to assess the roles of RgsC in stress responses. For oxidative stress test, hydrogen peroxide (5 mM), menadione (100 μM), and paraquat (100 μM) were added to the YG media after autoclaving. To assess cell wall stress, Congo red (100 μg/ml), calcofluor white (50 μg/ml), caspofungin (0.1 μg/ml) were added to the YG media after autoclaving. The production of gliotoxin (GT) was determined as described previously (Bok and Keller, 2004). The chloroform extracts were air-dried and resuspended in 100 ml of methanol. Ten micro liter aliquots of each sample were applied to a thin-layer chromatography (TLC) silica plate (Kiesel gel 60, E. Merck). The TLC plate was developed with toluene:ethyl acetae:formic acid (5:4:1, v/v/v) and GT standard was purchased from Sigma (USA). To test alternative nitrogen sources, MMG with nitrogen free salts was used as the base medium. Three grams per liter peptone and yeast extract, 6.0 g/l NaNO_3_, or 8.13 g/l proline was added, and these media were compared to MMG (NH_4_Cl).

### Enzyme assay

For catalase and superoxide dismutase (SOD) activity assays, protein was extracted as previous method (Jung et al., 2016). Catalase activity on gels was detected by ferricyanide-negative stain (Wayne and Diaz, 1986) and SOD activity was visualized by inhibition of the reduction of nitro blue tetrazolium (NBT, Sigma) according to the method of Beauchamp and Fridovich (1971).

### Insect virulence assay

The insect survival assay was performed as previously described with some modifications (Fuchs et al., 2010; Jung et al., 2016). Briefly, sixth instar *Galleria mellonella* were infected by injecting the fresh conidia (1 × 10^5^) into the last left pro-leg and incubated at 37°C in the dark for the duration of the experiment. Larvae were checked daily for survival and Kaplan-Meier survival curves were analyzed using the Log-Rank (Mantel-Cox) test for significance (*p* < 0.01).

### Microarray analysis

The synthesis of target cDNA probes and hybridization were performed using Agilent's Low Input Quick Amp WT Labeling Kit (Agilent Technology, USA) according to the manufacturer's instructions. Briefly, 100 ng total RNA was mixed with WT primer mix and incubated at 65°C for 10 min. cDNA master mix (5 × First strand buffer, 0.1 M DTT, 10 mM dNTP mix, RNase-Out, and MMLV-RT) was prepared and added to the reaction mixture. The samples were incubated at 40°C for 2 h, and then the RT and dsDNA synthesis reactions were terminated by incubating at 70°C for 10 min. The transcription master mix was prepared as directed by the manufacturer's protocol (4 × Transcription buffer, 0.1 M DTT, NTP mix, 50% PEG, RNase-Out, inorganic pyrophosphatase, T7-RNA polymerase, and Cyanine 3/5-CTP). Transcription of dsDNA was performed by adding the transcription master mix to the dsDNA reaction samples and incubating at 40°C for 2 h. Amplified and labeled cRNA was purified and labeled cRNA target was quantified. After checking labeling efficiency, each of cyanine 3-labeled and cyanine 5-labeled cRNA target were mixed, and fragmentation of cRNA was performed by adding 10 × blocking agent and 25 × fragmentation buffer and incubating at 60°C for 30 min. The fragmented cRNA was resuspended with 2 × hybridization buffer and directly pipetted onto assembled MYcroarray.com (*A*. *fumigatus* AF293) 30 K Microarray. The arrays hybridized at 57°C for 17 h using an Agilent Hybridization oven (Agilent Technology, USA). The hybridized microarrays were washed as per the manufacturer's washing protocol (Agilent Technology, USA). Hybridization images were analyzed by an Agilent DNA microarray Scanner (Agilent Technology, USA), and the data quantification was performed using Agilent Feature Extraction software 10.7 (Agilent Technology, USA). The average fluorescence intensity for each spot was calculated and local background was subtracted using Gene Pix Pro 6.0 (Axon Instruments, USA). Loess normalization and selection of fold-changed genes were performed using GenoWiz 4.0 (Ocimum biosolutions, India). The data is available in the Gene Expression Omnibus (GEO) at NCBI (the accession number is GSE83200).

## Results

### Summary of *A*. *fumigatus* RgsC

**Figure 1:**
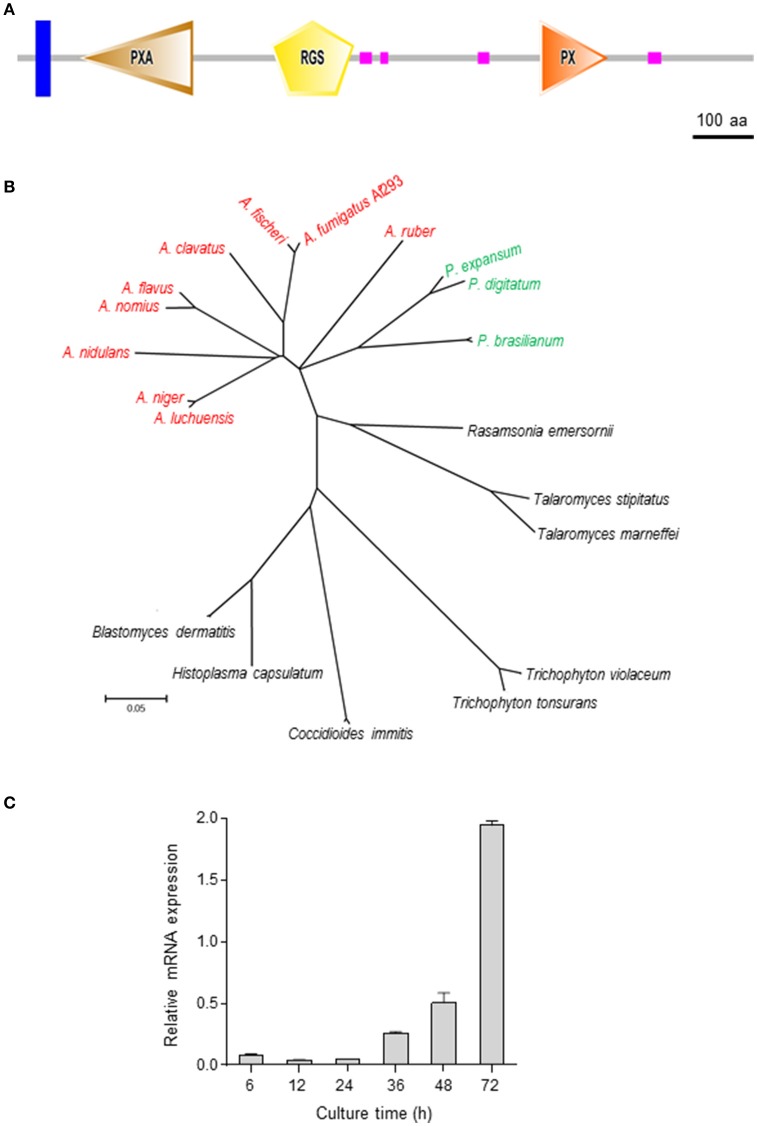
Summary of RgsC in *A*. *fumigatus* **(A)** Schematic presentation of the domain architecture of RgsC in *A*. *fumigatus* using SMART (http://smart.embl-heidelberg.de). **(B)** A phylogenetic tree of the RgsC-like proteins in various fungi was constructed based on the matrix of pair-wise distances between the sequences. **(C)** Expression of *rgsC* mRNA during the life cycle of WT.

The ORF of *rgsC* of *A*. *fumigatus* (AFUA_1G09040) consists of 3,718 bp nucleotides with 1 intron, predicted to encode a 1,216 aa length protein. As shown in Figure 1A, the domain structure of RgsC contains a transmembrane (31–53 aa), PXA (101–290 aa, E-value; 3.34e-27), RGS (419–556 aa, E-value; 8.34e-16), 4 low complexity, and 1 PX domain (863–976 aa, E-value; 2.78e-24). With these protein sequences, we further identified additional RgsC-like proteins in other fungi and carried phylogenetic analyses (Figure 1B). As presented, the *A*. *fumigatus* RgsC is closely related to that of *Aspergillus fischeri, Aspergillus clavatus, Aspergillus nomius, Aspergillus flavus, Aspergillus niger, A*. *nidulans*, and *Penicillium* spp., but phylogenetically distinct from RgsC of dimorphic fungi and dermatophytic fungi (Figure 1B). To characterize the *rgsC* gene, levels of *rgsC* mRNA at different time points in the life cycle were examined, and found to be low during the early vegetative growth and increased at the later phase of vegetative growth (Figure 1C).

### Roles of RgsC in asexual development

**Figure 2:**
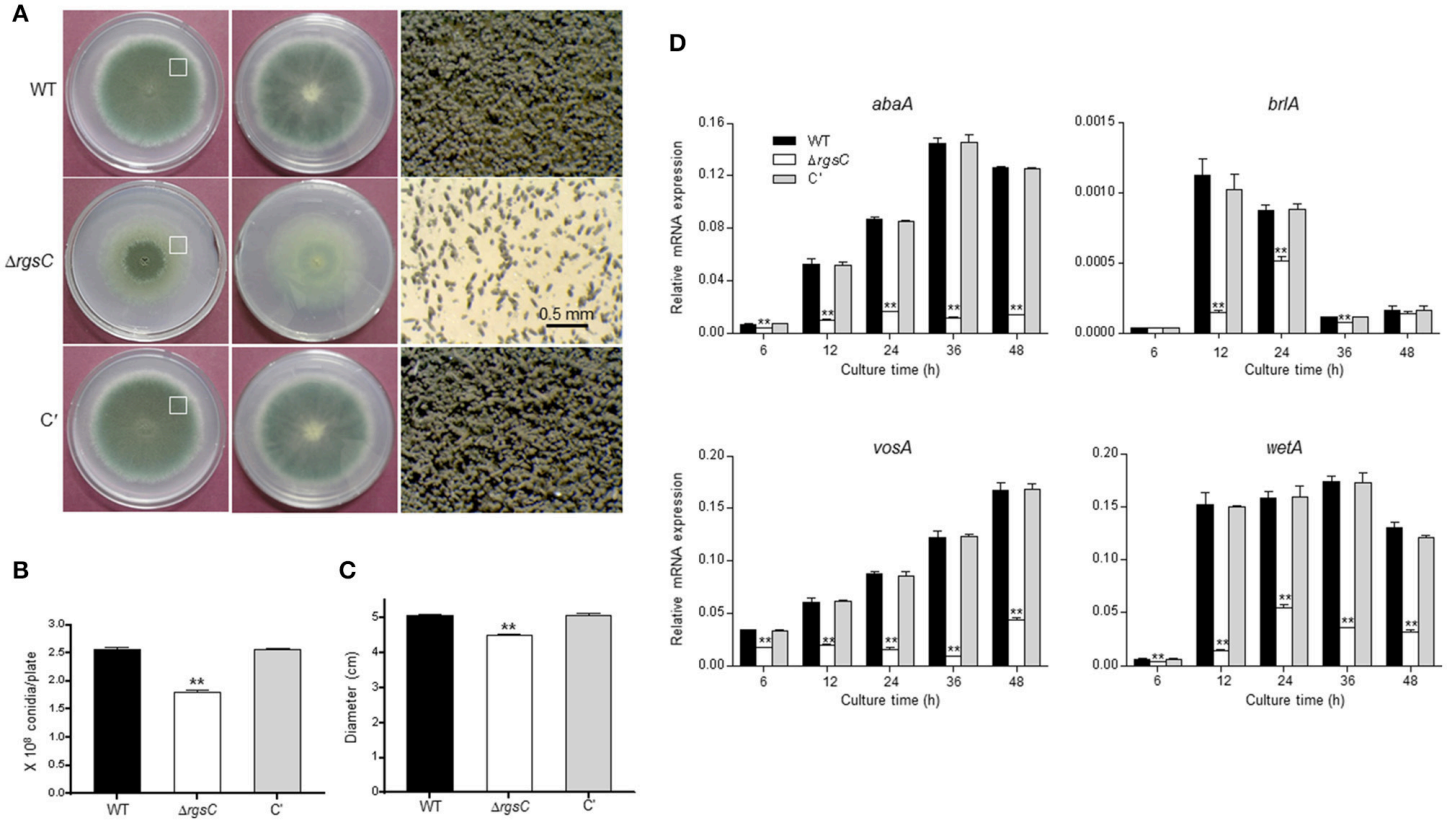
A role of RgsC in growth and development. **(A)** Colony photographs of WT (AF293), Δ*rgsC*, and complemented (C′) strains point-inoculated on solid MMY and grown for 3 days (Top: left; Bottom: middle panels). Enlarged photographs from the plate (indicated by the white box) are shown in the right panels with the bar indicating 0.5 mm. **(B)** Conidia numbers produced by each strain per plate. **(C)** Colony diameters of WT, Δ*rgsC*, and C′ strains. **(D)** mRNA levels of the asexual developmental regulators in WT, Δ*rgsC*, and C′ strains determined by quantitative real time PCR (qRT-PCR). Fungal cultures were done in liquid MMY and mRNA levels were normalized using the *ef1*α gene, according to the ΔΔCt method. Data are expressed as the mean ± standard deviation from three independent experiments. Student's *t*-test: ^*^p < 0.05; ^**^p < 0.01.

To characterize functions of *rgsC*, we generated the Δ*rgsC* mutant by replacing its ORF with the *A*. *nidulans argB*+ marker and we also generated complemented strains (C′) via re-introducing the wild type (WT) allele of *rgsC* to a deletion strain. Multiple Δ*rgsC* and C′ strains displaying identical phenotypes were isolated and further examined. When inoculated on solid medium, the *rgsC* deletion mutant formed a very distinct colony. The color of colony was very faint except a center region and the reverse side of colony was also light compared to WT and C' strains (Figure 2A). Moreover, whereas the colony edge of WT and C′ strains showed abundant conidiophores, the Δ*rgsC* mutant exhibited a very few number of conidiophores (Figure 2A, right panels). Conidia per plate further demonstrated that asexual spore production in the Δ*rgsC* mutant (1.8 × 10^8^ conidia/plate) was significantly decreased (*p* < 0.05) to a level that was only about 70% of WT and C′ strains (Figure 2B). Another noticeable change was that, the deletion of *rgsC* resulted in a significant reduction (about 85% of WT) in radial colony growth (Figure 2C). Further examination of mRNA levels of key asexual developmental regulators, *abaA, brlA, vosA*, and *wetA* in WT and Δ*rgsC* strains revealed a significantly reduced accumulation of these key developmental activators by the absence of *rgsC* (Figure 2D). As shown in Figure 2D, accumulation of *abaA* and *wetA* mRNAs increased from 12 h, peaked at 36 h, and decreased after 48 h post developmental induction in WT. Accumulation of *brlA* mRNA increased from 6 h, peaked at 12 h, and decreased after 24 h post developmental induction. However, the deletion of *rgsC* resulted in significantly low levels (*p* < 0.05) of these mRNAs at almost all times tested (Figure 2D). These results suggest that RgsC is necessary for proper growth and development in *A. fumigatus*.

### Elevated spore germination by Δ*rgsC*

**Figure 3:**
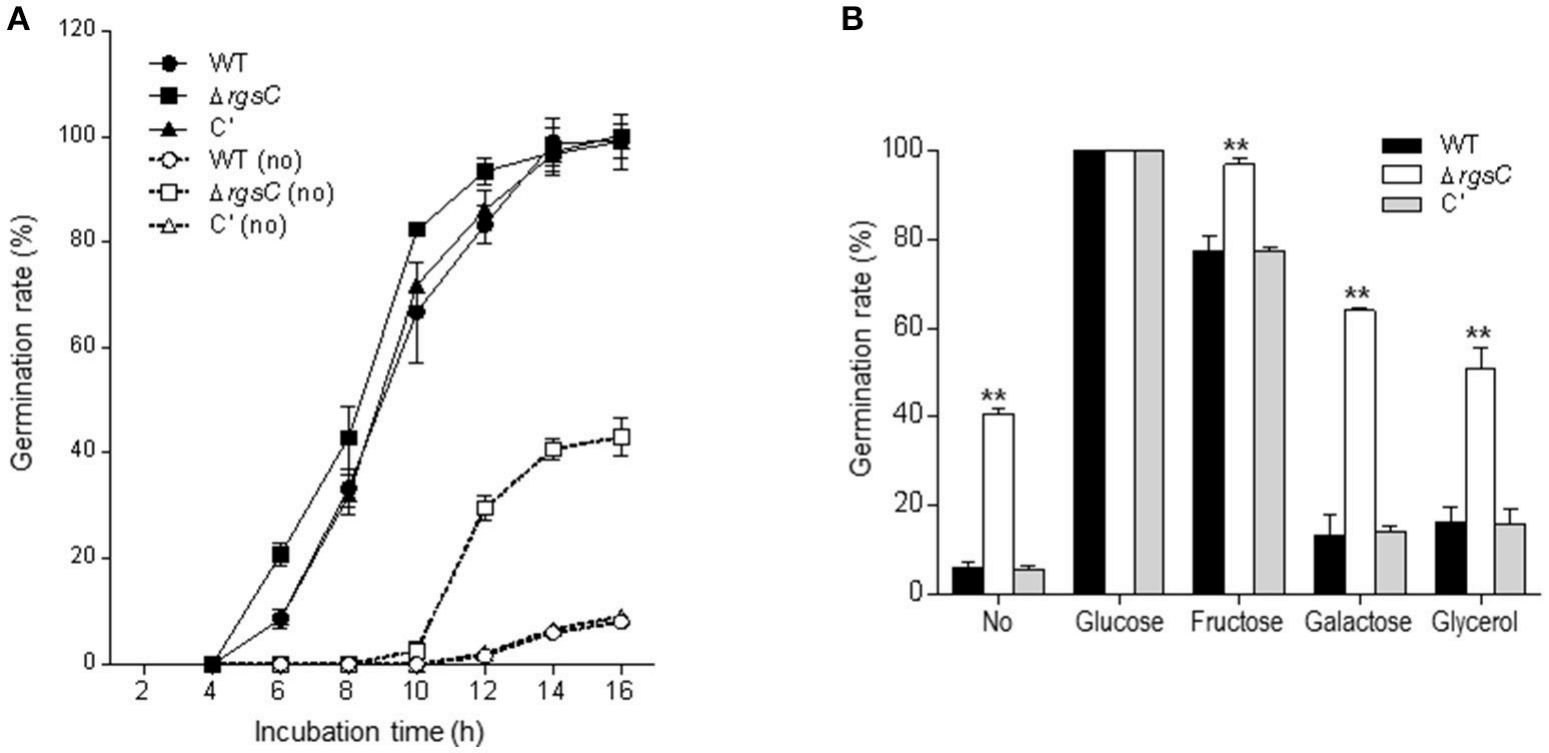
A role of RgsC in spore germination. **(A)** Kinetics of germ tube outgrowth in *A*. *fumigatus* strains when inoculated in liquid MMG at 37°C in the presence or absence (dashed line) of glucose. **(B)** Conidial germination in response to various carbon sources. Data are expressed as the mean ± standard deviation from three independent experiments. Student's *t*-test: ^*^p < 0.05; ^**^p < 0.01.

G protein signaling plays a positive role in spore germination (Fillinger et al., 2002; Lafon et al., 2005). If RgsC attenuates a G-protein signaling pathway activating germination, the absence of RgsC may result in elevated spore germination. To test this, we first inoculated conidia of WT, Δ*rgsC* mutant, and C′ strains in liquid MMY and analyzed the kinetics of germ tube emergence. As shown in Figure 3A, WT and C′ strains exhibited about 30% conidial germination at 8 h and near 100% germination at 14 h in liquid submerged culture. On the other hand, the Δ*rgsC* strain showed about 40% conidial germination at 8 h and near 100% germination at 12 h in liquid medium. To test further, we examined germination rates in the absence of external carbon source by inoculating conidia of WT, Δ*rgsC*, and C′ strains in liquid MMY (without glucose). After 16 h inoculation, whereas only about 10% of WT and C′ conidia showed germling formation, 40% of the Δ*rgsC* conidia germinated (Figure 3A), suggesting that RgsC may negatively regulate conidial germination potentially sensing the external carbon source. To investigate whether RgsC mediates sensing of carbon sources, germination of the Δ*rgsC* mutant conidia in comparison to that of WT and C′ strain conidia were monitored in the presence of various carbon sources. The germination rate was significantly elevated in the *rgsC* null mutant in all but glucose medium (Figure 3B), suggesting that RgsC is necessary for the proper control of spore germination in response to varying carbon sources.

### RgsC functions in oxidative stress responses

**Figure 4:**
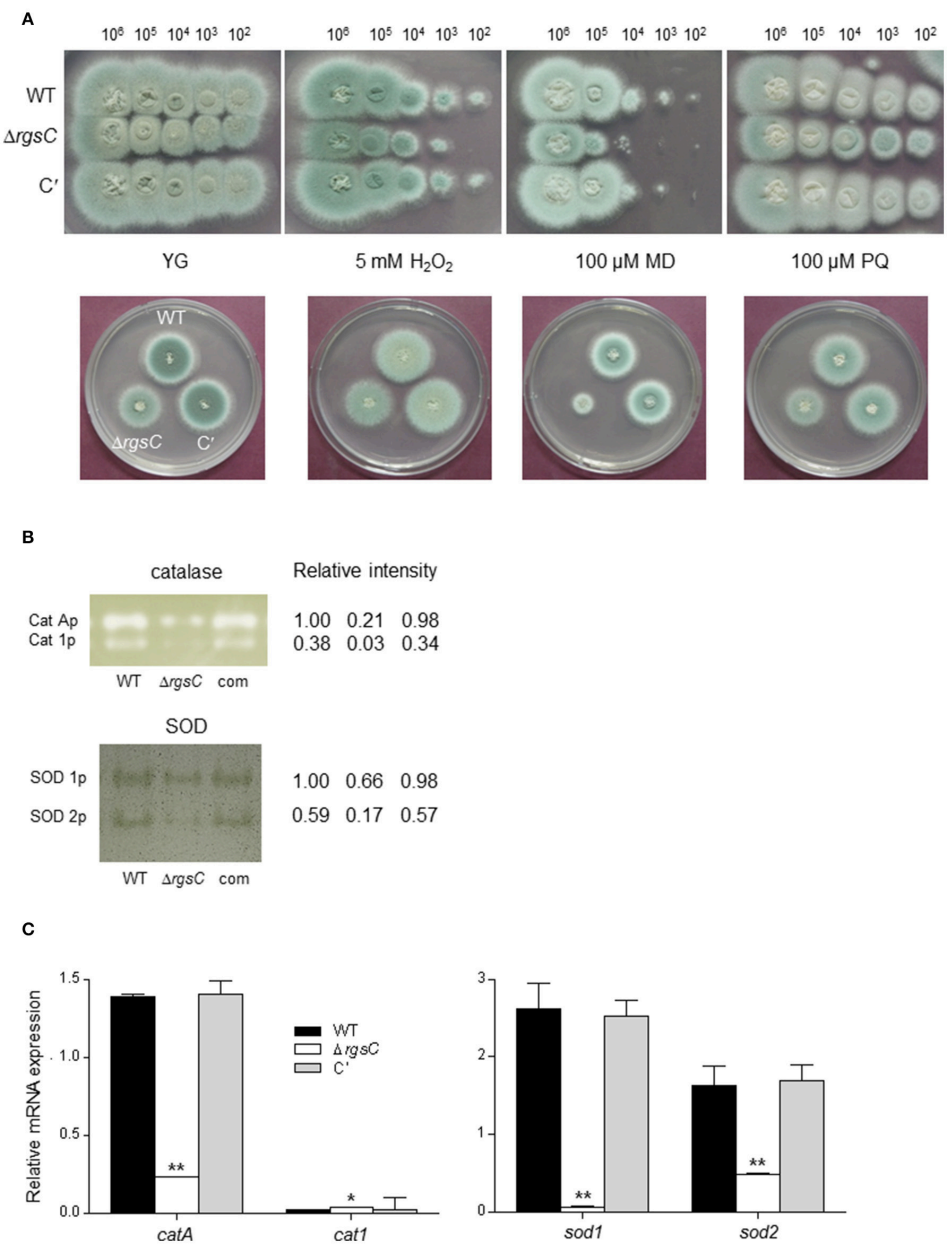
Oxidative stress tests. **(A)** Radial growth of WT, Δ*rgsC*, and C′ strains in presence of oxidative stressors H_2_O_2_, menadione (MD), or paraquat (PQ) at indicated concentrations following incubation at 37°C for 48 h. **(B)** Catalases and SODs activities of WT, Δ*rgsC*, and C′ strains shown in non-denaturing polyacrylamide gels. **(C)** Levels of catalase and SOD genes' mRNA in WT, Δ*rgsC*, and C′ strains analyzed by qRT-PCR. Statistical significance was determined by a Student's *t*-test: ^*^p < 0.05; ^**^p < 0.01.

To evaluate functions of RgsC in oxidative stress response, we incubated WT, Δ*rgsC*, and C′ strains in the presence of H_2_O_2_ and the reactive oxygen species (ROS) generating compounds menadione (MD) and paraquat (PQ). As shown in Figure 4A, while the Δ*rgsC* mutant was hypersensitive to MD, it exhibited a slightly reduced tolerance to H_2_O_2_ and PQ. To further investigate the role RgsC, we analyzed activities of the ROS detoxifying enzymes catalase and SOD. Activities of both conidia-specific (CatA) and mycelia-specific (Cat1) catalases were decreased about 5 to 10-fold in the Δ*rgsC* mutant compared to those of WT and C′ strains (Figure 4B). In *A*. *fumigatus*, four genes encoding SODs have been identified and SOD1 and SOD2 were shown to play a major role to detoxify intracellular superoxide anions (Lambou et al., 2010). As catalases activities, activities of SOD1 and SOD2 in the Δ*rgsC* mutant was only 60 and 20% of WT strain, respectively, suggesting that the reduced tolerance of the Δ*rgsC* mutant to oxidative stresses could be due to low detoxifying enzymes activities. We then examined whether the absence of *rgsC* affected mRNA levels of catalases and SODs. We found that mRNA levels of *catA, sod1*, and *sod2* were significantly decreased (*p* < 0.05) in the Δ*rgsC* mutant (Figure 4C). Taken together, these results suggest that RgsC is needed for protection against external oxidative stresses.

### RgsC is associated with cell wall stress responses

**Figure 5:**
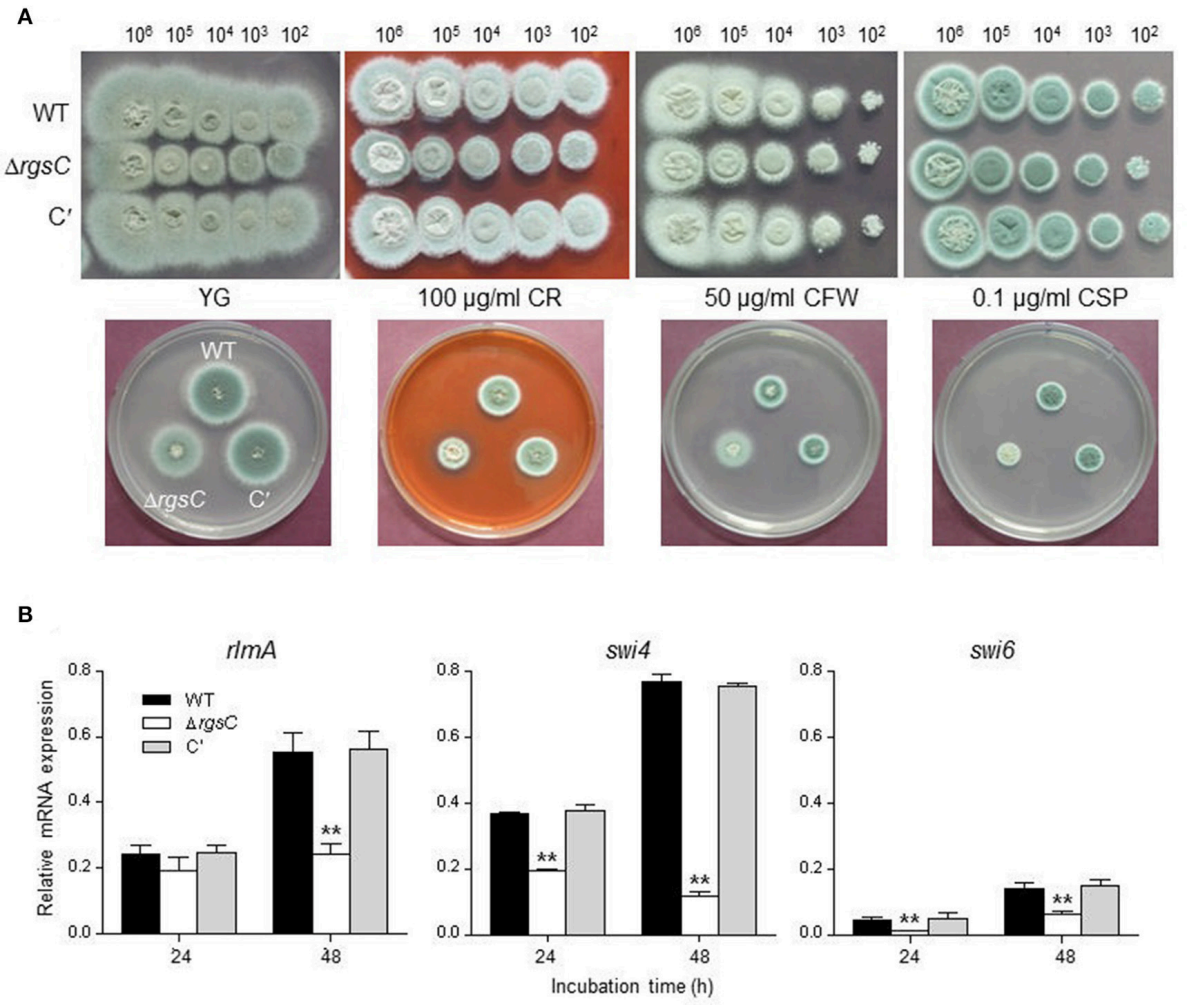
Cell wall stress tests. **(A)** Radial growth of WT, Δ*rgsC*, and C′ strains in presence of cell wall damaging agents Congo red (CR), calcofluor white (CFW), or caspofungin (CSP) at indicated concentrations following incubation at 37°C for 48 h. (**B**) Levels of cell wall integrity and biogenesis genes' mRNA in WT, Δ*rgsC*, and C′ strains analyzed by qRT-PCR. Statistical significance was determined by a Student's *t*-test: ^*^p < 0.05; ^**^p < 0.01.

To examine whether RgsC mediate cell wall stress response, the mutant were exposed to a variety of cell wall damaging compounds including Congo red (100 μg/ml), calcofluor white (50 μg/ml), and caspofungin (0.1 μg/ml). Growth of the Δ*rgsC* mutant was slightly inhibited by the tested compounds compared to that of WT and C′ strain (Figure 5A), suggesting cell wall biosynthesis and/or integrity may be affected by the deletion of *rgsC*. We further analyzed mRNA levels of the MADS-box transcription factor *rlmA* and APSES transcription factor *swi4* and *swi6* which regulate genes involved in cell wall integrity and biogenesis (Kim et al., 2010; Rocha et al., 2016). In the Δ*rgsC* mutant it appears that levels of all tested genes' mRNA decreased after 48 h compared to WT (Figure 5B). These results indicate that RgsC is associated with proper cell wall biogenesis and cell wall integrity.

### A role of RgsC in gliotoxin production and virulence

**Figure 6:**
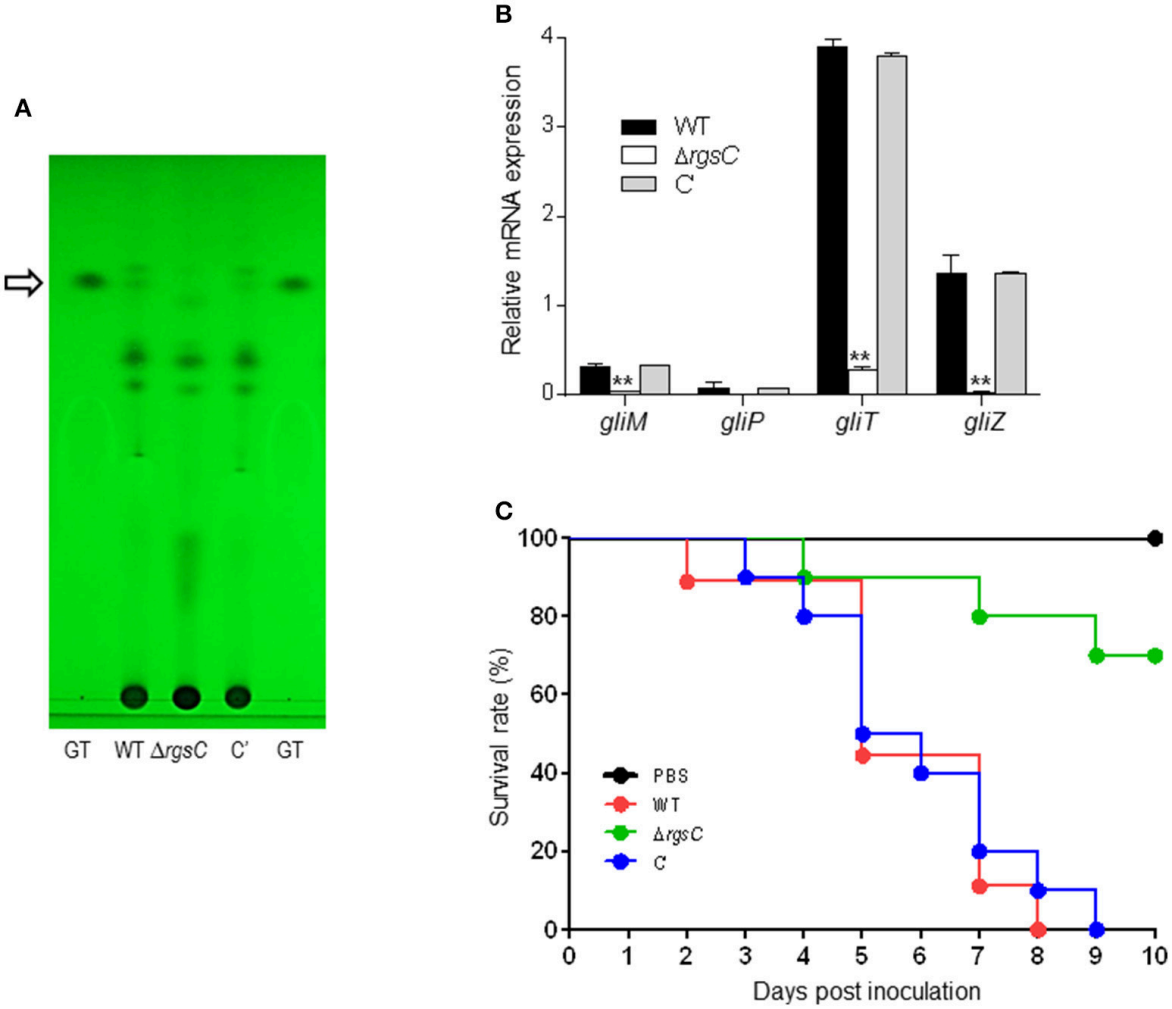
A role of RgsC in GT production and virulence. **(A)** Determination of GT production in WT, Δ*rgsC*, and C′ strains. The culture supernatant of each strain was extracted with chloroform and subjected to TLC. The arrow indicates the migration position for the GT standard. **(B)** qRT-PCR analysis of four *gli* cluster genes in WT, Δ*rgsC*, and C′ strains. Statistical differences between WT and mutant strains were evaluated with Student's unpaired *t*-test. ^*^p < 0.05; ^**^p < 0.01. **(C)** Survival curves of WT, Δ*rgsC*, and C′ strains measured using *G*. *mellonella* larvae. Note the significant differences (*p* < 0.002) between WT, Δ*rgsC*, and C′ strains.

In *A*. *fumigatus*, the gliotoxin (GT) production is partially regulated by the asexual developmental activator BrlA (Shin et al., 2015). The deletion of RGSs including FlbA, GprK, and Rax1 resulted in lowered *brlA* expression and GT production (Mah and Yu, 2006; Jung et al., 2016; Igbalajobi et al., 2017). As the deletion of *rgsC* resulted in significantly defective conidiation and *brlA* mRNA levels (Figure 2), we examined levels of GT by TLC. As shown in Figure 6A, the Δ*rgsC* mutant produced undetectable levels of GT. Then we analyzed mRNA levels of several key GT biosynthetic genes by qRT-PCR using total RNA of WT, mutant, and C' strains. The mRNA levels of the *gliM, gliT*, and *gliZ* genes were significantly lower (*p* < 0.05) in the Δ*rgsC* mutant than in WT and C′ strains (Figure 6B). We next examined the effect of RgsC on virulence using the *G*. *mellonella* larvae survival test. Conidia of WT, Δ*rgsC*, and C′ strains were inoculated in *G*. *mellonella* larvae, and the larvae survival rates were recorded as a function of time. The virulence of Δ*rgsC* strains in wax moth was significantly reduced compared to WT and C′ strains (Figure 6C). The Log-Rank test revealed that the survival curves of WT and Δ*rgsC* were significantly different (*p* < 0.002). These results indicate an important role of RgsC in proper production of GT and the virulence of the fungus.

### Transcriptome analysis

**Figure 7:**
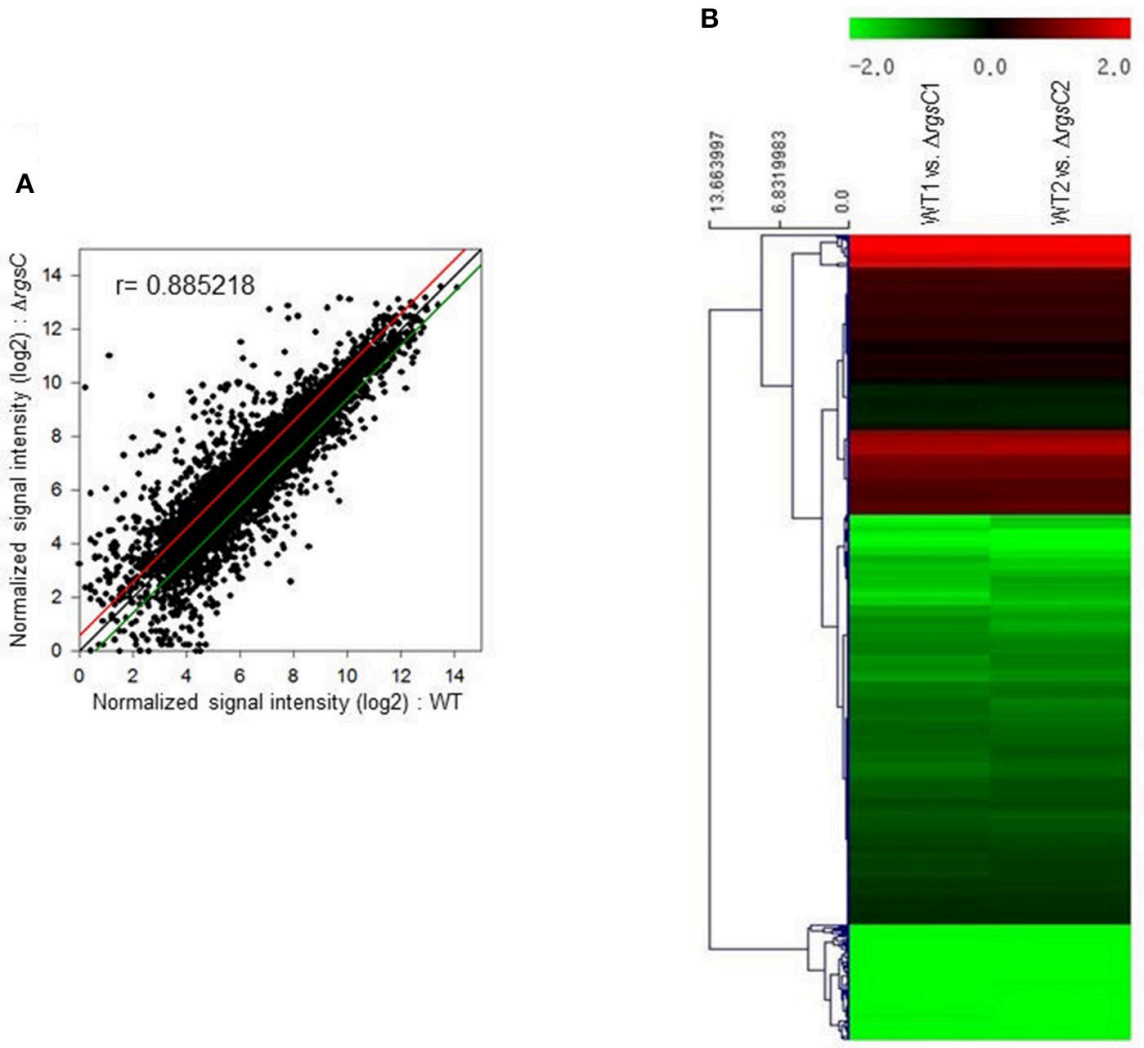
Genome-wide expression correlation between WT and Δ*rgsC* strains. **(A)** Linear fitted model showing the correlation between overall gene expression for WT and Δ*rgsC* strains. The correlation coefficient r is indicated. **(B)** Heat map illustration of expression level changes between WT and Δ*rgsC* strains.

**Figure 8:**
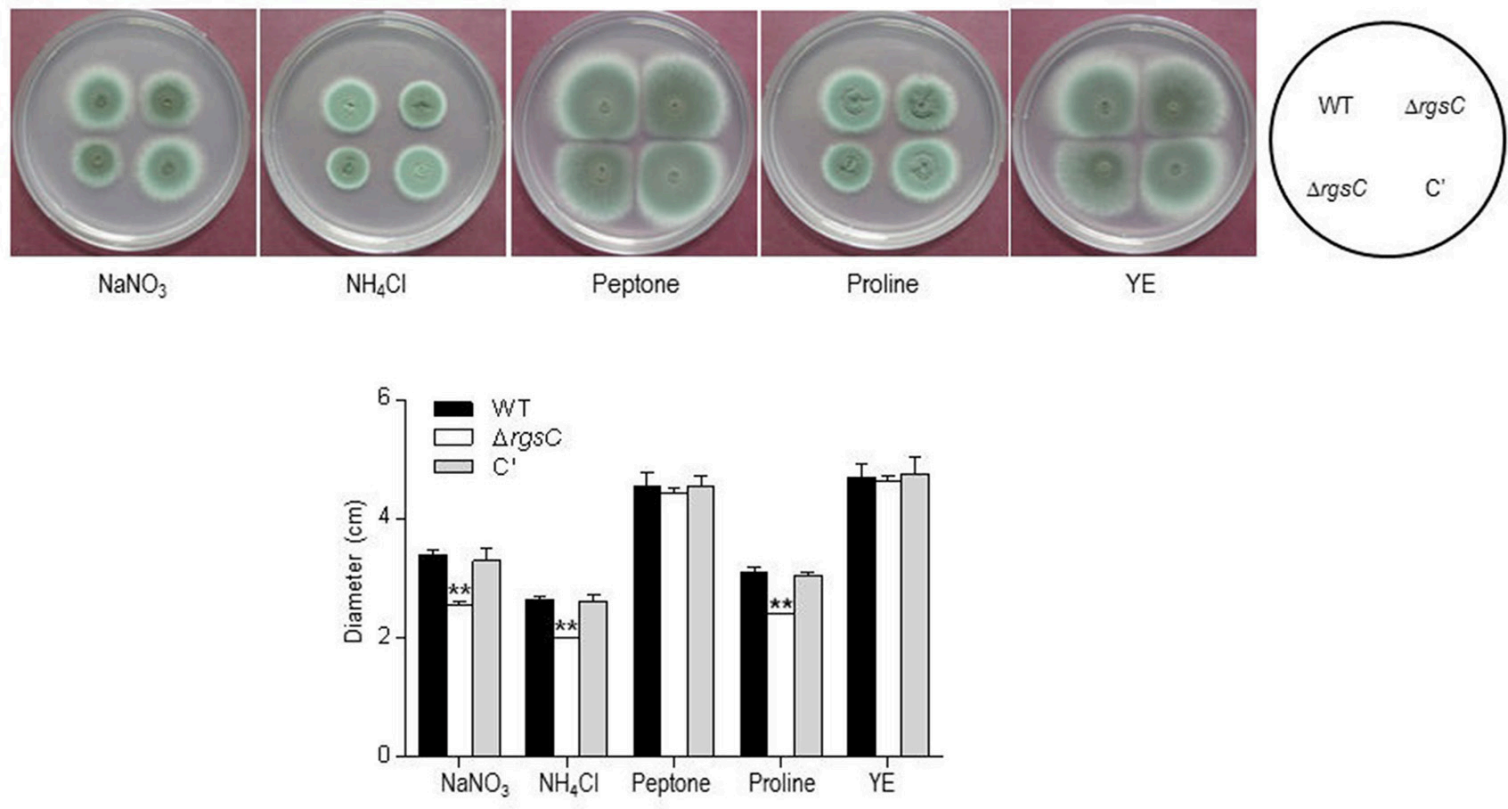
Effect of various nitrogen sources on growth of WT, Δ*rgsC*, and C′ strains. Note that growth of the mutant was significantly reduced in the presence of NaNO_3_, NH_4_Cl, and proline as nitrogen sources compared to WT and C′ strains. Statistical significance was determined by a Student's *t*-test: ^*^p < 0.05; ^**^p < 0.01.

To obtain a more comprehensive insight into the RgsC mediated processes in *A*. *fumigatus*, we performed microarray analysis using Δ*rgsC* and WT cells collected at 12 h post asexual-developmental induction. Two biological replicates showed a high level of correlation (*r* = 0.885, Figure 7A). As shown in Figure 7B, the hierarchical clustering heat map based on transcriptome analysis showed that a majority of genes are down-regulated in Δ*rgsC* strain compared to WT. Of the 8,608 probes, 384 genes (4.5%) showed at least 1.5-fold (*p* < 0.05) differentially expressed, in which 82 genes (1.0%) were up-regulated and 302 genes (3.5%) were down-regulated (Supplementary Table 2). Table 1 lists the genes with increase in expression at least 2.0-fold (*p* < 0.01) following the deletion of *rgsC*. The highest up-regulated gene was predicted to encode a conserved hypothetical protein (AFUA_8G06430), with salicylate hydroxylase (AFUA_2G00770) identified as the up-regulated known gene with the maximum fold change in mutant relative to WT. The existence of transcripts corresponding to the conserved hypothetical proteins were first confirmed by qRT-PCR on the same RNA used for the microarray library construction (data not shown). Most of the down-regulated genes were related to nitrogen transport (Table 2), including small oligopeptide transporter (AFUA_2G15240), high affinity nitrate transporter NrtB (AFUA_1G17470), MFS peptide transporter (AFUA_1G12240), ammonium transporter MeaA (AFUA_2G05880), and nitrate transporter CrnA (AFUA_1G12850). These findings led us to test a role for the RgsC in nitrogen source sensing. The Δ*rgsC* mutant was grown on a variety of nitrogen sources such as, NaNO_3_, NH_4_Cl, peptone, proline, and yeast extract. As shown in Figure 8, while there were no differences in growth on organic nitrogen sources, growth of the Δ*rgsC* mutant was significantly (*p* < 0.01) restricted with NaNO_3_, NH_4_Cl, and proline as a nitrogen source, suggesting that RgsC may play a role in inorganic and simple nitrogen sensing.

**Table 1:** Up-regulated genes in Δ*rgsC* relative to WT (> 2.0-fold, *p* < 0.01).

**Probe set ID**	**Gene symbol**	**Product**	**Log_2_FC**	**FDR *p*-value**
5751203	AFUA_8g06430	Conserved hypothetical protein	8.156	0.003
5751027	AFUA_8g05700	Conserved hypothetical protein	4.102	0.003
5733645	AFUA_2g00770	Salicylate hydroxylase	2.993	0.003
5741485	AFUA_4g08180	Hypothetical protein	2.491	0.003
5744036	AFUA_5g06680	4-aminobutyrate transaminase GatA	2.022	0.003
5732310	AFUA_1g12570	RNA binding protein Ligatin/Tma64, putative	1.928	0.004
5739542	AFUA_3g12600	Beta-glucosidase, putative	1.825	0.003
5739346	AFUA_3g11640	Homoserine dehydrogenase	1.507	0.000
5730512	AFUA_1g02890	dUTPase (Dut), putaive	1.416	0.003
5742433	AFUA_4g12870	Methylmalonate-semialdehyde dehydrogenase	1.386	0.001
5741718	AFUA_4g09220	Flavin-binding monooxygenase-like protein	1.278	0.003
5738761	AFUA_3g08960	Epoxide hydrolase, putative	1.250	0.002

**Table 2:** Down-regulated genes in Δ*rgsC* relative to WT (> 2.0-fold, *p* < 0.01).

**Probe set ID**	**Gene symbol**	**Product**	**Log_2_FC**	**FDR *p*-value**
5730612	AFUA_1G03360	Conserved hypothetical protein	−7.247	0.001
5736469	AFUA_2G15240	Small oligopeptide transporter, OPT family	−6.274	0.002
5745980	AFUA_6G00640	Integral membrane protein	−5.420	0.001
5745818	AFUA_5G14940	Cell surface metalloreductase (FreA), putative	−4.888	0.003
5733082	AFUA_1G16060	Conserved hypothetical protein	−4.315	0.001
5734611	AFUA_2G05180	NF-X1 finger and helicase domain protein	−4.120	0.003
5736439	AFUA_2G15110	C2H2 finger domain protein, putative	−4.025	0.003
5737902	AFUA_3G03760	Hypothetical protein	−3.879	0.004
5733419	AFUA_1G17470	High affinity nitrate transporter NrtB	−3.792	0.002
5732250	AFUA_1G12240	MFS peptide transporter, putative	−3.759	0.001
5751177	AFUA_8G06350	Esterase family protein	−3.707	0.004
5746940	AFUA_6G07060	Alpha/beta hydrolase family protein, putative	−3.444	0.003
5749748	AFUA_7G06260	Zinc-containing alcohol dehydrogenase, putative	−3.173	0.001
5732767	AFUA_1G14660	Regulator of secondary metabolism LaeA	−3.101	0.004
5743540	AFUA_5G03269	Conserved hypothetical protein	−3.080	0.002
5744277	AFUA_5G07730	Conserved hypothetical protein	−3.029	0.003
5747107	AFUA_6G07790	Hypothetical protein	−3.011	0.001
5747297	AFUA_6G08650	Hypothetical protein	−2.927	0.001
5734775	AFUA_2G05880	Ammonium transporter MeaA	−2.926	0.002
5742705	AFUA_4G14230	MFS transporter, putative	−2.804	0.004
5737930	AFUA_3G03940	2,3-diketo-5-methylthio-1-phosphopentane	−2.763	0.004
5735540	AFUA_2G10890	VPS9 domain protein, putative	−2.742	0.005
5741172	AFUA_4G06620	Glu/Leu/Phe/Val dehydrogenase	−2.621	0.003
5732370	AFUA_1G12850	Nitrate transporter CrnA	−2.587	0.002
5749755	AFUA_7G06290	Pfs, NACHT, and Ankyrin domain protein	−2.526	0.001
5747764	AFUA_6G10720	Alpha-ketoglutarate-dependent taurine	−2.521	0.002
5737424	AFUA_3G01620	Ankyrin and HET domain protein	−2.307	0.004
5743121	AFUA_5G01290	Zinc-binding oxidoreductase, putative	−2.265	0.001
5745897	AFUA_6G00280	NmrA-like family protein	−2.110	0.004
5732823	AFUA_1G14910	Endosomal SPRY domain protein, putative	−2.081	0.002
5735099	AFUA_2G08660	Conserved hypothetical protein	−2.080	0.001
5743241	AFUA_5G01900	Heat shock transcription factor Hsf1, putative	−2.021	0.004
5743118	AFUA_5G01272	C6 transcription factor, putative	−1.948	0.002
5744784	AFUA_5G10020	Sensor histidine kinase/response regulator	−1.929	0.002
5750200	AFUA_8G00830	Conserved hypothetical protein	−1.763	0.001
5742984	AFUA_5G00720	GNAT family acetyltransferase, putative	−1.744	0.003
5733109	AFUA_1G16160	C6 transcription factor, putative	−1.641	0.003
5748131	AFUA_6G12440	Conserved hypothetical protein	−1.612	0.003
5733448	AFUA_1G17610	Hypothetical protein	−1.591	0.004
5749290	AFUA_7G04290	Amino acid permease (Gap1), putative	−1.587	0.004
5733076	AFUA_1G16030	Conserved hypothetical protein	−1.575	0.004
5734316	AFUA_2G03900	Acetamidase/Formamidase family protein	−1.511	0.002
5750769	AFUA_8G04370	GPI anchored protein, putative	−1.469	0.002
5739657	AFUA_3G13100	Conserved hypothetical protein	−1.454	0.001
5750005	AFUA_7G08530	Conserved hypothetical protein	−1.432	0.001
5739136	AFUA_3G10660	Hydroxymethylglutaryl-CoA synthase Erg13	−1.353	0.003
5730340	AFUA_1G02080	Conserved hypothetical protein	−1.295	0.002
5749943	AFUA_7G08231	Hypothetical protein	−1.268	0.001
5749669	AFUA_7G05880	Conserved hypothetical protein	−1.167	0.003
5731901	AFUA_1G10630	S-adenosylmethionine synthetase	−1.154	0.002
5732164	AFUA_1G11900	PQ loop repeat protein	−1.143	0.001
5745798	AFUA_5G14845	RING-finger domain protein, putative	−1.141	0.004
5733064	AFUA_1G16000	Serine/threonine protein kinase, putative	−1.104	0.001
5730263	AFUA_1G01700	Conserved serine-rich protein	−1.100	0.004
5749754	AFUA_7G06280	Conserved hypothetical protein	−1.081	0.004
5745754	AFUA_5G14670	Conserved hypothetical protein	−1.075	0.004
5744934	AFUA_5G10790	Oxidoreductase, short chain	−1.024	0.001
5748718	AFUA_7G00700	Aldo-keto reductase (AKR13), putative	−1.020	0.001
5738815	AFUA_3G09240	CAIB/BAIF family enzyme	−1.016	0.001
5740145	AFUA_3G15250	MFS drug efflux transporter, putative	−1.014	0.005
5745824	AFUA_5G14950	Conserved serine-proline rich protein	−1.005	0.002

## Discussion

All life forms are able to sense and respond to various internal and external stimuli, and numerous signaling pathways play an important roles during the cellular processes. G-protein signaling is conserved in all eukaryotes that sense and transmit signals into the cells to amplify appropriate responses (Dohlman et al., 1996). Basic units of the G-protein signaling system typically include a G protein-coupled receptor (GPCR), regulators of G protein signaling (RGS), a heterotrimeric G protein composed of α, β, and γ subunits, and a variety of effectors (Li et al., 2007). RGS proteins are a family of multifunctional signaling regulators having the capacity to bind to activated Gα subunits. Canonical RGS proteins stimulate the intrinsic GTPase activity of the cognate Gα subunits and lead to deactivation of Gα subunits and termination of signaling (De Vries et al., 2000; Siderovski and Willard, 2005). In various fungi, RGSs have been shown to regulate morphogenesis, differentiation, reproduction, toxin production, and virulence (Lengeler et al., 2000; Mah and Yu, 2006; Zhang et al., 2011; Jung et al., 2016; Igbalajobi et al., 2017). Consequently, elucidation of the regulatory mechanisms of RGS proteins is expected to provide a basis for identifying novel targets for controlling human pathogenic fungi.

The five RGS proteins defined in *A*. *nidulans* can be grouped into three clades (A, B, and C), where the clade A can further be divided into the sub-clades A-I and A-II. Sub-clade A-I contains 10 RGS proteins and members of A-I all have multiple conserved functional domains, such as, PXA, PX, and Nexin_C (Wang et al., 2013). Based on the domain organization, RgsC of *A*. *fumigatus* may belong to the sub-clade A-I. Although it has been speculated that the RgsC-type fungal RGS proteins might function in coordinating G-protein signaling, hyphal extension, nuclear transmission, and organelle transport (Han et al., 2004b), exact function of these proteins are not clear yet.

In the present report, we show several experimental evidence that the RgsC plays a crucial role in governing vegetative growth and asexual development in *A*. *fumigatus*. The absence of *rgsC* results in profound defects in vegetative growth, asexual sporulation, and lowered expression of key asexual developmental regulators (Figure 2). Moreover, overall, the germination rate of the Δ*rgsC* mutant was significantly higher than that of WT and C′ strains in the absence of carbon source and in the presence of carbon sources other than glucose (Figure 3). It was shown that germination can be induced by various carbon sources by activation of the cAMP/PKA pathway in *A*. *nidulans* (Fillinger et al., 2002), implying that RgsC might function in proper control of the cAMP/PKA pathway and spore germination.

*A. fumigatus* has five catalases (Calera et al., 1997; Paris et al., 2003a) and four SODs (Holdom et al., 2000; Flückiger et al., 2002; Lambou et al., 2010) that can be associated with detoxification of ROS. We investigated the sensitivity of the Δ*rgsC* mutant against ROS generating compounds and found that the Δ*rgsC* conidia were significantly more sensitive to compounds tested than the WT and C′ conidia (Figure 4A). Activities of catalases (CatA and Cat1) and SOD (SOD1 and 2) were drastically decreased in the Δ*rgsC* mutant (Figure 4B). The mRNA levels of *catA, sod1*, and *sod2* in the Δ*rgsC* conidia were significantly lower than those of the WT and C′ conidia (Figure 4C). Previous studies demonstrated that the deletion of a conidial catalase *catA* resulted in increased susceptibility of conidia to H_2_O_2_, but disruptions of the either mycelial catalases (*cat1* or *cat 2*) did not affect sensitivity to H_2_O_2_ (Calera et al., 1997; Paris et al., 2003a,b). These finding suggest that CatA plays a major role in detoxification of H_2_O_2_. Sod1 and Sod2 were highly expressed in conidia during growth and both of the Δ*sod1* and Δ*sod2* mutants showed hypersensitivity to MD (Lambou et al., 2010). Taken together, RgsC may positively regulate the expression of the key ROS detoxifying enzymes catalases and SODs, conferring proper oxidative stress response.

The Δ*rgsC* mutant showed increased susceptibility to cell wall disturbing agents such as, CR, CFW, and CSF (Figure 5A). We have investigated mRNA expressions of genes related to cell wall integrity and biogenesis. The cell wall integrity pathway is the primary signaling cascade that controls the synthesis of the fungal cell wall and is highly dependent on the RlmA transcription factor (Rocha et al., 2016). Loss-of-function of *rlmA* leads to the altered cell wall organization, tolerance to cell wall perturbing agents, and expression of genes encoding cell wall-related proteins (Rocha et al., 2016). The cell cycle transcription factor SBF (Swi4 and Swi6) interacts with the protein kinase C/MAP kinase pathway, which functions in the control of cell wall assembly, thus loss of SBF function leads to a weakened wall (Igual et al., 1996). In the Δ*rgsC* mutant, levels of *rlmA, swi4*, and *swi6* mRNA decreased after 48 h compared to WT (Figure 5B), suggesting that RgsC may take part in the regulation of cell wall integrity signaling and cell wall assembly pathway. In susceptibility test against azole antifungal agents, there was no significant difference between WT and mutant strains may due to azole antifungal drugs inhibit ergosterol synthetic enzyme (Sheehan et al., 1999) (Supplementary Figure 2).

Biogenesis of gliotoxin (GT) requires activities of the *gli* gene cluster composed of 13 genes in *A*. *fumigatus* (Gardiner and Howlett, 2005). Several studies indicate that GT plays a direct role in aspergillosis virulence in immunocompromised individuals (Gardiner et al., 2005; Lewis et al., 2005; Spikes et al., 2008). In GT biosynthesis, the *gliM* gene is predicted to encode an *o*-methyltransferase (Cramer et al., 2006). GliP, a multimodular nonribosomal peptide synthetase, makes the diketopiperazine scaffold of GT (Balibar and Walsh, 2006). The GT oxidoreductase GliT protects the fungus against exogenous GT and is essential for GT biosynthesis (Schrettl et al., 2010; Brakhage, 2013). The *gliZ* gene controls expression of the remaining genes the *gli* gene cluster (Bok et al., 2006; Scharf et al., 2012). We found that GT production and expression of GT biosynthetic genes in the Δ*rgsC* mutant were severely reduced compared to WT and C′ strain (Figure 6), suggesting that RgsC plays a positive role in GT synthesis, likely by conferring proper activation of the asexual developmental regulator *brlA*. GT suppresses the immune response of *G*. *mellonella* larvae by inhibiting the action of haemocytes and thus renders the larvae susceptible (Reeves et al., 2004). The mortality level of the Δ*rgsC* mutant in wax moth larvae was significantly (*p* < 0.002) reduced compared to the WT and C′ strains (Figure 6C), which in part may be due to the defective production of GT in the Δ*rgsC* mutant. Collectively, the data indicate that RgsC-mediated modulation/attenuation of signal transduction pathway(s) is important for proper control of GT biogenesis and virulence of *A. fumigatus*. While we do not know the target heterotrimeric G protein(s) and/or other signaling elements modulated by RgsC, as RgsC is highly conserved in many pathogenic ascomycete fungi including species of *Blastomyces, Histoplasma*, and *Coccidioides* but not found in human, it might be an excellent target for the development of novel antifungal drugs.

Of the 8,608 probes, 384 genes were found to be differentially expressed by the absence of RgsC, and most of them were down-regulated. Intriguingly, most of nitrogen transport-related genes were down-regulated by Δ*rgsC*, including small oligopeptide transporter, high affinity nitrate transporter NtrB, MFS peptide transporter, ammonium transporter MeaA, nitrate transporter CrnA, amino acid permease Gap1, and MFS drug efflux transporter (Table 2). The results imply that RgsC is needed for proper expression of these genes, and the Δ*rgsC* mutant might not sense external nitrogen sources effectively. To confirm this, we tested growth of mutant on various nitrogen sources. Growth of the Δ*rgsC* mutant was significantly (*p* < 0.01) reduced with NaNO_3_, NH_4_Cl, and proline as the nitrogen source (Figure 8). These results suggest that the Δ*rgsC* mutant fails to sense and/or transport external inorganic and simple nitrogen sources effectively. The key regulator of secondary metabolism LaeA (AFUA_1G14660) was also down-regulated in the Δ*rgsC* mutant. LaeA represents a global regulator of secondary metabolism and the *A*. *fumigatus* Δ*laeA* mutant is unable to produce GT (Cramer et al., 2006). Collectively, defective GT production in the Δ*rgsC* mutant might result from reduced expression of *laeA* and *brlA*.

## Author contributions

KS and JY conceived and supervised the study; KS and JY designed experiments; YK, IH, and KS performed experiments; KS and JY analyzed data; YK, IH, JY, and KS wrote the manuscript.

### Conflict of interest statement

The authors declare that the research was conducted in the absence of any commercial or financial relationships that could be construed as a potential conflict of interest. The reviewer JM and handling Editor declared their shared affiliation.

## Supplementary material

Supplementary Figure1 Confirmation of WT, Δ*rgsC*, and complemented (C′) strains. **(A)** Schematic illustration of the *rgsC* regions in WT, Δ*rgsC*, and complemented (C′) strains. **(B)** PCR amplicons for the three strains. Lane M, molecular weight marker. **(C)** The HindIII digestion pattern of individual amplicon. While the WT and C′ amplicons are cut into two fragments, the Δ*rgsC* amplicon remains uncut.

Supplementary Figure2 Clear E-test growth inhibition ellipses for ketoconazole and voriconazole. About 106 conidia were inoculated in YG media containing appropriate supplements and cultured at 37°C for 24 h.
