# Supplementary figures and images for: Characteristics of a Regulator of G-Protein Signaling (RGS) rgsC in Aspergillus fumigatus
Source: Front Microbiol. 2017 Oct 23;8:2058. doi: 10.3389/fmicb.2017.02058 (PMC5660106; doi:10.3389/fmicb.2017.02058)

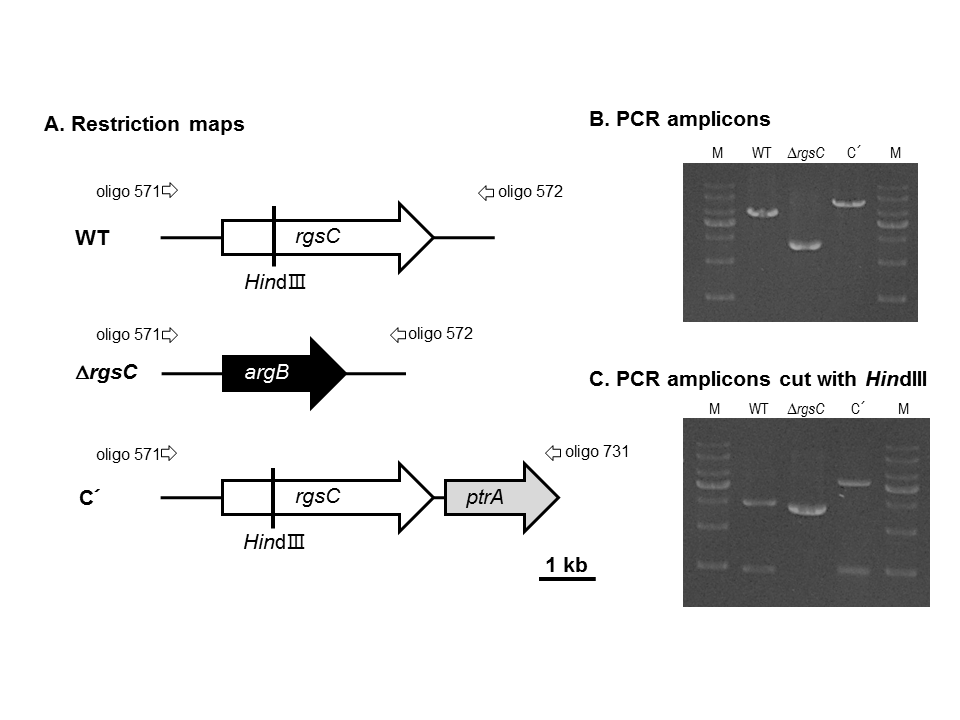

Supplement: Supplementary Figure1 — Confirmation of WT, ΔrgsC, and complemented (C′) strains. (A) Schematic illustration of the rgsC regions in WT, ΔrgsC, and complemented (C′) strains. (B) PCR amplicons for the three strains. Lane M, molecular weight marker. (C) The HindIII digestion pattern of individual amplicon. While the WT and C′ amplicons are cut into two fragments, the ΔrgsC amplicon remains uncut. [file Image1.TIF]

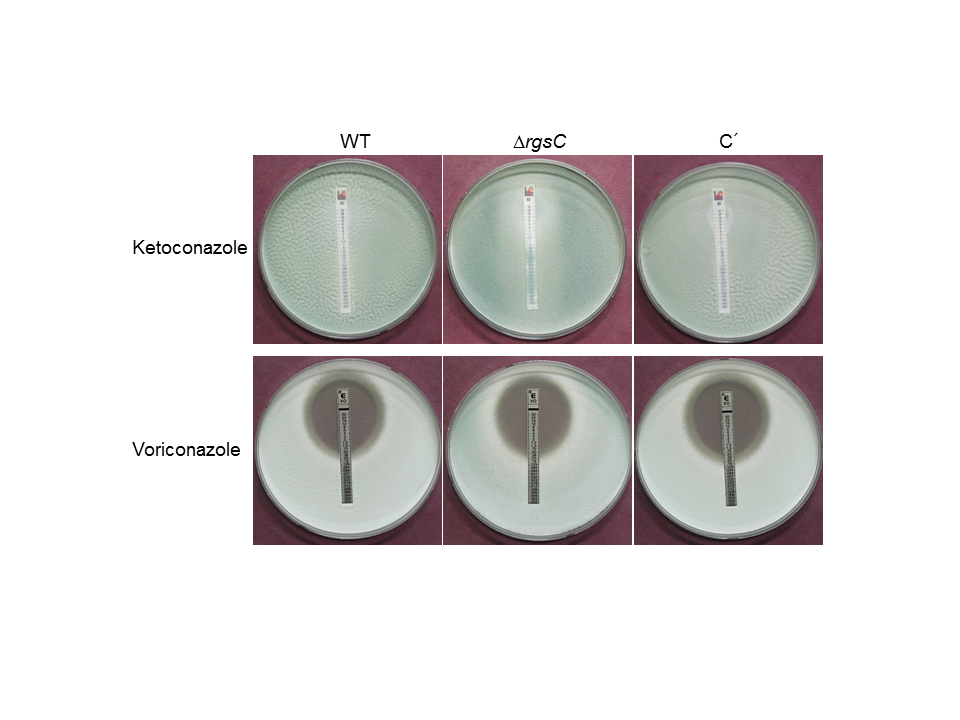

Supplement: Supplementary Figure2 — Clear E-test growth inhibition ellipses for ketoconazole and voriconazole. About 106 conidia were inoculated in YG media containing appropriate supplements and cultured at 37°C for 24 h. [file Image2.TIF]
