# Supplementary material for: Characteristics of a Regulator of G-Protein Signaling (RGS) rgsC in Aspergillus fumigatus
Source: Front Microbiol. 2017 Oct 23;8:2058. doi: 10.3389/fmicb.2017.02058 (PMC5660106; doi:10.3389/fmicb.2017.02058)
Supplement: Supplementary file 3 [file Table1.DOC]

**TABLE S1 | Oligonucleotides used in this study.**

| **Name** | **Sequence (5'→3')^a^** | **Purpose** |
| --- | --- | --- |
| oligo346  oligo347  oligo303  oligo304  oligo305  oligo306  oligo307  oligo308  oligo256  oligo257  oligo420  oligo421  oligo422  oligo423  oligo426  oligo427  oligo275  oligo276  oligo277  oligo278  oligo260  oligo261  oligo262  oligo263  oligo689  oligo690  oligo691  oligo692  oligo693  oligo694  oligo271  oligo272  oligo214  oligo215  oligo567  oligo568  oligo569  oligo570  oligo571  oligo572  oligo781  oligo782  oligo783  oligo730  oligo731 | CCATGTGTGTCGAGTCCTTC  GAACGTACAGCAACAGTCTGG  GCTACCACTCTGCATCCTCA  TACGAGCTCCAGCATGATTC  ACGGCAGGAAGTTGTCTTCT'  CTGTCAGCGACTTGTTGGAT  ATGAACCAGCCAGGTACTCC  GTTGGTCCCTTGTGGAAAGT  TTCCAAGCAGAGCTTGTCAC  CCAGGTTCTTTGCACTTGAA  GTGCAGCAGTCAGGGATCTA  ATCGAACTTGGTGCTTACCC  CTTGTCCATGCAATAGCCTA  GGTCTGTACCATGCCTGTTG  CAGCTCCTGATATCAAGCCA'  CGCTTGGTATTTGCACCTTA  AGCCACGGCTCTTCTACAAT  GTTCTTCACCACGGGAGATT  ATTCCAGAGGAGAAGCAGGA  GAGCTCTCCAATCACACGAA  TCATTGCTGTCCTCCGTGGTG  GGTCGTTGCCCTTGATGTTCC  GGTCAGTTCCAGCCTCTTCTTG  CTTCTCCACAGCCTTCCAGTTG  AAACCCCTGTGAATGCAGAC  CCCCTTGAGATGAAAGGTGA  CGATCTGTACCCCAACGAGT  TTCTGGAACTTTGCCAGCTT  ACTCCACCATCCAGTTCCAG  TCCGAGTATCCCTCGATGTC  AAATCCATCACATCCACCCT  GGTTGTTCATGGTCAGTTGC  ACGCTGACGATGAGGCTGATGCTC  AGGATTCGCTTGACTGTCTTCTCC  CCGAGAAAGTAGCATCCGTAGT  *TCCGTCGTGATGACGGTGATATTG*AGAAGTAGGAGCTATTCGAGGTC  *ACTCGCAAGAGCTAAGGTCTGTC*AGGCAATCAGACGAACTCGCAT  CGTAACCTCAAGGGTGTCACAG  ACGAGTCAAGTAGTGCTTTCG  ACTGACCGTTCCCTTTAGGC  GTCGTTGCTGCTTCACCAGT  *AAACAAAGATGCAAGAGCGGC*GTCTGATTGCCTCAGGCGAA  CGCCTGAGGCAATCAGACGA*GCCGCTCTTGCATCTTTGTTT*'  GCCGCTCTTGCATCTTTGTTT  AATTGATTACGGGATCCCATTGG | 5' *ef1* for qRT-PCR normalization  3' *ef1* for qRT-PCR normalization  5' *abaA* for qRT-PCR  3' *abaA* for qRT-PCR  5' *wetA* for qRT-PCR  3' *wetA* for qRT-PCR  5' *vosA* for qRT-PCR  3' *vosA* for qRT-PCR  5' *brlA* for qRT-PCR  3' *brlA* for qRT-PCR  5' *swi4* for qRT-PCR  3' *swi4* for qRT-PCR  5' *swi6* for qRT-PCR  3' *swi6* for qRT-PCR  5' *rlmA* for qRT-PCR  3' *rlmA* for qRT-PCR  5' *cat1* for qRT-PCR  3' *cat1* for qRT-PCR  5' *catA* for qRT-PCR  3' *catA* for qRT-PCR  5' *sod1* for qRT-PCR  3' *sod1* for qRT-PCR  5' *sod2* for qRT-PCR  3' *sod2* for qRT-PCR  5' *gliP* for qRT-PCR  3' *gliP* for qRT-PCR  5' *gliM* for qRT-PCR  3' *gliM* for qRT-PCR  5' *gliT* for qRT-PCR  3' *gliT* for qRT-PCR  5' *gliZ* for qRT-PCR  3' *gliZ* for qRT-PCR  5' *AnargB* marker  3' *AnargB* marker  5' flanking region of *rgsC*  5' *rgsC* with *AnargB* tail  3' *rgsC* with *AnargB* tail  3' flanking region of *rgsC*  5' nested of *rgsC*  3' nested of *rgsC*  5' *rgsC* for complementation  3' *rgsC* with *ptrA* for complementation  5' *ptrA* with *rgsC* for complementation  5' of *ptrA*  3' nested of *ptrA* |

^a^ Tail sequence is in italic.
