# Supplementary material for: Characteristics of a Regulator of G-Protein Signaling (RGS) rgsC in Aspergillus fumigatus
Source: Front Microbiol. 2017 Oct 23;8:2058. doi: 10.3389/fmicb.2017.02058 (PMC5660106; doi:10.3389/fmicb.2017.02058)
Supplement: Supplementary file 4 [file Table2.DOC]

**TABLE S2 | Differentially expressed genes in Δ*rgsC* relative to WT (> 1.5-fold, *p* < 0.05).**

| **Probe Set ID** | **Gene Symbol** | **Product** | **Fold change** | **FDR *p*-value** |
| --- | --- | --- | --- | --- |
| 5751203  5750519  5750044  5734197  5751027  5733645  5741485  5737233  5745357  5740700  5747826  5733936  5740669  5744036  5733154  5732310  5739542  5749126  5731252  5744061  5749132  5732712  5739346  5750264  5730512  5739607  5735043  5742433  5741235  5733696  5741718  5738761  5738835  5744644  5732745  5738932  5736195  5732425  5730674  5740568  5732080  5738939  5747436  5742251  5743221  5741769  5740891  5748200  5749532  5745451  5748388  5745078  5742429  5741028  5744744  5749341  5741440  5732967  5746350  5750691  5736666  5733788  5737609  5744537  5738322  5738358  5748275  5747706  5736234  5735268  5732960  5735175  5731676  5744041  5734273  5731510  5741604  5746202  5732149  5748205  5734044  5745131  5731037  5749353  5747306  5748287  5737894  5746685  5751052  5746534  5735787  5738261  5739594  5743412  5750305  5748885  5732756  5730675  5745395  5746617  5738505  5738419  5735952  5748933  5730863  5744148  5736368  5733321  5730442  5735978  5749293  5730780  5744244  5739530  5747100  5731103  5741222  5748862  5740845  5750582  5733829  5745336  5732954  5744920  5743991  5731112  5744553  5730248  5732064  5738749  5731653  5731928  5733538  5738919  5735958  5749088  5738104  5745485  5735494  5730561  5743334  5743042  5738657  5747458  5741241  5741461  5735237  5751174  5734276  5733402  5735732  5733198  5732873  5737503  5741177  5750082  5733180  5735104  5736893  5750251  5749843  5740373  5733078  5737121  5744778  5747638  5743636  5744396  5732554  5748847  5733912  5746631  5737455  5738967  5737468  5740768  5746275  5745903  5733118  5733535  5750880  5746855  5746069  5745824  5740145  5738815  5748718  5744934  5742996  5743491  5748727  5745578  5734813  5732797  5739199  5736645  5732578  5737262  5745754  5749754  5743194  5742352  5730263  5733064  5742706  5744559  5731418  5745248  5745798  5732164  5742981  5731901  5739890  5749669  5740706  5750470  5730522  5751011  5732241  5736278  5738847  5733328  5750477  5743261  5742679  5746841  5749943  5736689  5730340  5749726  5742028  5744255  5739136  5734605  5737634  5737518  5748091  5741214  5737419  5750005  5747417  5739657  5737641  5745795  5750769  5749343  5737019  5746113  5731222  5731308  5734316  5749115  5744078  5738106  5741678  5745569  5733076  5749290  5733448  5743646  5749445  5733819  5748131  5751339  5744635  5733109  5750624  5750467  5743196  5746005  5737022  5742984  5750200  5748327  5740775  5742682  5739266  5737856  5744784  5743118  5750984  5740290  5743090  5746300  5742989  5743241  5748394  5736986  5742265  5735099  5732823  5745897  5732325  5751069  5740004  5738746  5743121  5744019  5737424  5735065  5740530  5734127  5735690  5748124  5745956  5745210  5732432  5733013  5730314  5747764  5749755  5732370  5733434  5740116  5741172  5740522  5733067  5735540  5737930  5732619  5736163  5743911  5743018  5742705  5734775  5747297  5731386  5751365  5745826  5746284  5747107  5744277  5746290  5750370  5743540  5732767  5748400  5732395  5735341  5749748  5743045  5733915  5745761  5735991  5748397  5731744  5731187  5746940  5745693  5738554  5750078  5751177  5737163  5732457  5746427  5732250  5733419  5751244  5737902  5748747  5736439  5750473  5734611  5733082  5730490  5731954  5751128  5751180  5745818  5739180  5741273  5745980  5736469  5730612 | AFUA_8G06430  AFUA_8G02310  AFUA_8G00130  AFUA_2G03340  AFUA_8G05700  AFUA_2G00770  AFUA_4G08180  AFUA_3G00770  AFUA_5G12690  AFUA_4G03450  AFUA_6G11020  AFUA_2G02110  AFUA_4G03325  AFUA_5G06680  AFUA_1G16330  AFUA_1G12570  AFUA_3G12600  AFUA_7G02520  AFUA_1G06490  AFUA_5G06790  AFUA_7G02510  AFUA_1G14380  AFUA_3G11640  AFUA_8G01160  AFUA_1G02890  AFUA_3G12870  AFUA_2G08340  AFUA_4G12870  AFUA_4G07020  AFUA_2G01030  AFUA_4G09220  AFUA_3G08960  AFUA_3G09340  AFUA_5G09400  AFUA_1G14530  AFUA_3G09850  AFUA_2G14090  AFUA_1G13090  AFUA_1G03640  AFUA_4G02880  AFUA_1G11520  AFUA_3G09880  AFUA_6G09210  AFUA_4G11840  AFUA_5G01820  AFUA_4G09420  AFUA_4G04270  AFUA_6G12840  AFUA_7G05280  AFUA_5G13110  AFUA_6G13770  AFUA_5G11430  AFUA_4G12840  AFUA_4G05900  AFUA_5G09850  AFUA_7G04500  AFUA_4G07940  AFUA_1G15610  AFUA_6G03340  AFUA_8G04000  AFUA_2G16140  AFUA_2G01440  AFUA_3G02470  AFUA_5G08890  AFUA_3G06830  AFUA_3G07050  AFUA_6G13230  AFUA_6G10440  AFUA_2G14280  AFUA_2G09510  AFUA_1G15580  AFUA_2G09080  AFUA_1G09590  AFUA_5G06710  AFUA_2G03720  AFUA_1G08840  AFUA_4G08715  AFUA_6G02660  AFUA_1G11810  AFUA_6G12870  AFUA_2G02590  AFUA_5G11710  AFUA_1G05490  AFUA_7G04550  AFUA_6G08680  AFUA_6G13310  AFUA_3G03740  AFUA_6G04890  AFUA_8G05790  AFUA_6G04230  AFUA_2G12160  AFUA_3G06510  AFUA_3G12810  AFUA_5G02650  AFUA_8G01340  AFUA_7G01410  AFUA_1G14620  AFUA_1G03630  AFUA_5G12870  AFUA_6G04560  AFUA_3G07720  AFUA_3G07360  AFUA_2G13020  AFUA_7G01670  AFUA_1G04560  AFUA_5G07160  AFUA_2G14820  AFUA_1G17040  AFUA_1G02580  AFUA_2G13110  AFUA_7G04300  AFUA_1G04170  AFUA_5G07560  AFUA_3G12530  AFUA_6G07740  AFUA_1G05790  AFUA_4G06940  AFUA_7G01290  AFUA_4G04070  AFUA_8G02550  AFUA_2G01630  AFUA_5G12620  AFUA_1G15560  AFUA_5G10660  AFUA_5G06480  AFUA_1G05830  AFUA_5G08965  AFUA_1G01650  AFUA_1G11460  AFUA_3G08900  AFUA_1G09460  AFUA_1G10780  AFUA_2G00330  AFUA_3G09780  AFUA_2G13030  AFUA_7G02310  AFUA_3G05760  AFUA_5G13270  AFUA_2G10690  AFUA_1G03090  AFUA_5G02320  AFUA_5G00980  AFUA_3G08450  AFUA_6G09315  AFUA_4G07040  AFUA_4G08050  AFUA_2G09350  AFUA_8G06340  AFUA_2G03730  AFUA_1G17390  AFUA_2G11840  AFUA_1G16570  AFUA_1G15140  AFUA_3G02010  AFUA_4G06640  AFUA_8G00300  AFUA_1G16490  AFUA_2G08690  AFUA_2G17220  AFUA_8G01070  AFUA_7G06675  AFUA_4G00960  AFUA_1G16050  AFUA_3G00270  AFUA_5G10010  AFUA_6G10120  AFUA_5G03730  AFUA_5G08235  AFUA_1G13630  AFUA_7G01240  AFUA_2G01990  AFUA_6G04640  AFUA_3G01790  AFUA_3G09980  AFUA_3G01850  AFUA_4G03730  AFUA_6G03040  AFUA_6G00310  AFUA_1G16190  AFUA_2G00320  AFUA_8G04890  AFUA_6G06640  AFUA_6G02030  AFUA_5G14950  AFUA_3G15250  AFUA_3G09240  AFUA_7G00700  AFUA_5G10790  AFUA_5G00770  AFUA_5G03030  AFUA_7G00720  AFUA_5G13740  AFUA_2G06070  AFUA_1G14790  AFUA_3G10930  AFUA_2G16060  AFUA_1G13750  AFUA_3G00900  AFUA_5G14670  AFUA_7G06280  AFUA_5G01650  AFUA_4G12440  AFUA_1G01700  AFUA_1G16000  AFUA_4G14210  AFUA_5G09000  AFUA_1G07450  AFUA_5G12230  AFUA_5G14845  AFUA_1G11900  AFUA_5G00710  AFUA_1G10630  AFUA_3G14120  AFUA_7G05880  AFUA_4G03470  AFUA_8G02050  AFUA_1G02940  AFUA_8G05610  AFUA_1G12210  AFUA_2G14460  AFUA_3G09390  AFUA_1G17070  AFUA_8G02090  AFUA_5G01990  AFUA_4G14100  AFUA_6G06570  AFUA_7G08231  AFUA_2G16240  AFUA_1G02080  AFUA_7G06130  AFUA_4G10720  AFUA_5G07620  AFUA_3G10660  AFUA_2G05150  AFUA_3G02585  AFUA_3G02080  AFUA_6G12240  AFUA_4G06890  AFUA_3G01590  AFUA_7G08530  AFUA_6G09120  AFUA_3G13100  AFUA_3G02610  AFUA_5G14840  AFUA_8G04370  AFUA_7G04520  AFUA_2G17820  AFUA_6G02230  AFUA_1G06350  AFUA_1G06850  AFUA_2G03900  AFUA_7G02460  AFUA_5G06860  AFUA_3G05800  AFUA_4G09060  AFUA_5G13715  AFUA_1G16030  AFUA_7G04290  AFUA_1G17610  AFUA_5G03790  AFUA_7G04930  AFUA_2G01590  AFUA_6G12440  AFUA_8G07090  AFUA_5G09370  AFUA_1G16160  AFUA_8G02720  AFUA_8G02060  AFUA_5G01690  AFUA_6G00740  AFUA_2G17830  AFUA_5G00720  AFUA_8G00830  AFUA_6G13510  AFUA_4G03760  AFUA_4G14110  AFUA_3G11280  AFUA_3G03570  AFUA_5G10020  AFUA_5G01272  AFUA_8G05460  AFUA_4G00600  AFUA_5G01200  AFUA_6G03140  AFUA_5G00740  AFUA_5G01900  AFUA_6G13790  AFUA_2G17620  AFUA_4G11920  AFUA_2G08660  AFUA_1G14910  AFUA_6G00280  AFUA_1G12620  AFUA_8G05850  AFUA_3G14665  AFUA_3G08890  AFUA_5G01290  AFUA_5G06580  AFUA_3G01620  AFUA_2G08470  AFUA_4G02710  AFUA_2G03020  AFUA_2G11630  AFUA_6G12410  AFUA_6G00550  AFUA_5G12035  AFUA_1G13120  AFUA_1G15800  AFUA_1G01960  AFUA_6G10720  AFUA_7G06290  AFUA_1G12850  AFUA_1G17550  AFUA_3G15110  AFUA_4G06620  AFUA_4G02650  AFUA_1G15990  AFUA_2G10890  AFUA_3G03940  AFUA_1G13940  AFUA_2G13930  AFUA_5G06070  AFUA_5G00870  AFUA_4G14230  AFUA_2G05880  AFUA_6G08650  AFUA_1G07250  AFUA_8G07190  AFUA_5G14970  AFUA_6G03070  AFUA_6G07790  AFUA_5G07730  AFUA_6G03090  AFUA_8G01630  AFUA_5G03269  AFUA_1G14660  AFUA_6G13810  AFUA_1G12970  AFUA_2G09860  AFUA_7G06260  AFUA_5G01000  AFUA_2G02000  AFUA_5G14710  AFUA_2G13170  AFUA_6G13800  AFUA_1G09880  AFUA_1G06160  AFUA_6G07060  AFUA_5G14320  AFUA_3G07980  AFUA_8G00280  AFUA_8G06350  AFUA_3G00440  AFUA_1G13210  AFUA_6G03690  AFUA_1G12240  AFUA_1G17470  AFUA_8G06590  AFUA_3G03760  AFUA_7G00805  AFUA_2G15110  AFUA_8G02070  AFUA_2G05180  AFUA_1G16060  AFUA_1G02780  AFUA_1G10930  AFUA_8G06100  AFUA_8G06360  AFUA_5G14940  AFUA_3G10850  AFUA_4G07190  AFUA_6G00640  AFUA_2G15240  AFUA_1G03360 | conserved hypothetical protein  aldehyde dehydrogenase ALDH  hypothetical protein  DUF652 domain protein  conserved hypothetical protein  salicylate hydroxylase  hypothetical protein  conserved hypothetical protein  dihydroxyacetone kinase (DakA), putative  NmrA-like family protein  3-hydroxyisobutyrate dehydrogenase  MFS monosaccharide transporter, putative  conserved hypothetical protein  4-aminobutyrate transaminase GatA  ribosome assembly protein Noc2, putative  RNA binding protein Ligatin/Tma64, putative  beta-glucosidase, putative  conserved serine-rich protein  conserved hypothetical protein  conserved hypothetical protein  conserved hypothetical protein  3-ketoacyl-acyl carrier protein reductase,  homoserine dehydrogenase  tartrate dehydrogenase, putative  dUTPase (Dut), putaive  GTPase activating protein (Gyp3), putative  conserved hypothetical protein  methylmalonate-semialdehyde dehydrogenase,  mitochondrial cytochrome b2-like, putative  1-aminocyclopropane-1-carboxylate deaminase,  flavin-binding monooxygenase-like protein  epoxide hydrolase, putative  PDCD2_C domain protein, putative  carbonyl reductase, putative  general amidase, putative  DNA mismatch repair protein Msh2, putative  extragenic suppressor of the bimD6 mutation  anthranilate synthase multifunctional protein  conserved hypothetical protein  conserved hypothetical protein  fungal specific transcription factor, putative  exosome complex exonuclease Rrp6, putative  SIR2 family histone deacetylase, putative  glyoxylate reductase  DUF221 domain protein, putative  carbonic anhydrase, putative  thymidylate synthase  kinesin family protein  conserved hypothetical protein  conserved hypothetical protein  C6 finger domain protein, putative  quinone oxidoreductase, putative  class II aldolase/adducin domain protein  conserved hypothetical protein  tRNA exportin, putative  ATP phosphoribosyltransferase His1, putative  oxidoreductase, 2-nitropropane dioxygenase  oxidoreductase, zinc-binding dehydrogenase  conserved hypothetical protein  acetyl-CoA acetyltransferase, putative  DEAD/DEAH box helicase, putative  mitochondrial carrier protein, putative  amidase family protein  homoaconitase LysF  aspartate-semialdehyde dehydrogenase  WSC domain protein, putative  nitrilase family protein (Nit3), putative  conserved hypothetical protein  kinesin family protein (KlpA), putative  conserved hypothetical protein  acetylserotonin methytransferase-like protein  arginine N-methyltransferase (Rmt2), putative  conserved hypothetical protein  DUF89 domain protein  peptidyl-prolyl cis-trans isomerase  guanylate kinase  conserved hypothetical protein  conserved hypothetical protein  conserved hypothetical protein  ABC iron exporter Atm1, putative  aspartyl-tRNA synthetase Dps1, putative  conserved hypothetical protein nuclear distribution protein NudE  histone deacetylase complex subunit (Hos4),  serine/threonine protein kinase, putative  phosphotyrosyl phosphatase activator  26S proteasome non-ATPase regulatory subunit  protein kinase, putative  CaaX prenyl proteinase Rce1  conserved hypothetical protein  RING finger protein  conserved hypothetical protein  conserved hypothetical protein  transcriptional corepressor of histone genes  hypothetical protein  MFS sugar transporter, putative  UPF0183 domain protein  Fibronectin type III domain protein  GPI anchored serine-threonine rich protein  NAD+ kinase, putative  37S ribosomal protein S9  conserved hypothetical protein  mitochondrial metallochaperone Sco1, putative  mitochondrial dicarboxylate carrier, putative  MFS transporter Fmp42, putative  urease Ure  DUF636 domain protein  conserved hypothetical protein  D-lactate dehydrogenase, putative  lipase, putative  cytochrome c  RhoGAP and Fes/CIP4 domain protein  fungal specific transcription factor, putative  capsular associated protein, putative  sensor histidine kinase/response regulator,  peroxisomal biogenesis factor (PEX11), putative  GPI anchored serine-rich protein  sphingolipid desaturase, putative  conserved hypothetical protein  mucin family signaling protein Msb2, putative  MFS peptide transporter, putative  MSF1 domain protein  CorA family metal ion transporter, putative  hypothetical protein  pentatricopeptide repeat protein  Sad1/UNC domain protein  TBP associated factor (Mot1), putative  cytochrome c oxidase assembly protein (Pet191),  C6 finger domain protein, putative  1,3-beta-glucanosyltransferase Bgt1  tubulin-specific chaperone c, putative  NIF domain protein  glycine cleavage system T protein  beta-alanine synthase, putative  conserved hypothetical protein  phenylalanyl-tRNA synthetase alpha subunit  adenine phosphoribosyltransferase 1  C6 transcription factor (Fcr1), putative  pH signal transduction protein PalH  MFS phosphate transporter, putative  mitochondrial integral membrane protein  conserved hypothetical protein  MFS multidrug transporter, putative  hypothetical protein  hypothetical protein  aspartic-type endopeptidase (CtsD), putative  phosphoinositide phosphatase (Sac1), putative  endo-beta-1,6-glucanase, putative  conserved hypothetical protein  Ctr copper transporter family protein  conserved hypothetical protein  transcriptional corepressor Cyc8, putative  MIND kinetochore complex component Mtw1,  mitochondrial phosphate carrier protein (Mir1),  cytochrome P450 monooxygenase, putative  acid sphingomyelinase, putative  conserved hypothetical protein  short chain dehydrogenase/reductase, putative  nuclear distribution protein NudE  C2H2 transcription factor (AmdX), putative  sterol desaturase, putative  COG3602 family protein  6-phosphofructokinase alpha subunit  conserved serine-proline rich protein  GPI-anchored cell wall beta-1,3-endoglucanase  extracellular serine-threonine rich protein  zinc-binding oxidoreductase ToxD, putative  conserved hypothetical protein  NUDIX family hydrolase, putative  D-isomer specific 2-hydroxyacid dehydrogenase  phytase, putative  signal recognition particle 14kD protein,  hydrolase, alpha/beta fold family, putative  isoflavone reductase family protein  conserved hypothetical protein  porphyromonas-type peptidyl-arginine deiminase  dienelactone hydrolase  MFS multidrug transporter, putative  serine carboxypeptidase (CpdS), putative  extracellular cell wall glucanase Crf1/allergen  sterol delta 5,6-desaturase, putative  conserved hypothetical protein  FK506 suppressor Sfk1, putative  aminotransferase, putative  conserved serine-proline rich protein  MFS drug efflux transporter, putative  CAIB/BAIF family enzyme  aldo-keto reductase (AKR13), puatative  oxidoreductase, short chain  integral membrane protein, putative  C6 transcription factor, putative  conserved hypothetical protein  protein phosphatase, putative  mRNA splicing factor (Prp1/Zer1), putative  mitochondrial inheritance component mdm12  bZIP transcription factor (MeaB), putative  conserved hypothetical protein  C2H2 transcription factor (Rpn4), putative  alpha-amylase, putative  conserved hypothetical protein  conserved hypothetical protein  bZIP transcription factor JlbA/IDI-4  sulfate transporter family protein  conserved serine-rich protein  serine/threonine protein kinase, putative  conserved hypothetical protein  sister chromatid cohesion protein (Eso1),  mitochondrial GTP/GDP transporter Ggc1,  MYND domain protein (SamB), putative  RING-finger domain protein, putative  PQ loop repeat protein  GABA permease, putative  S-adenosylmethionine synthetase  hypothetical protein  conserved hypothetical protein  tannase, putative  conserved hypothetical protein  mitotic check point protein (Bub2), putative  cell wall glucanase (Scw11), putative  NADH oxidase  oxidoreductase, short-chain  AMMECR1 family protein  FYVE domain protein, putative  nucleotide-sugar transporter, putative  BYS1 domain protein, putative  glutathione S-transferase, putative  conserved hypothetical protein  hypothetical protein  cell division control protein Cdc25, putative  conserved hypothetical protein  plasma membrane protein Pth11-like, putative  conserved hypothetical protein  DUF1212 domain membrane protein  hydroxymethylglutaryl-CoA synthase Erg13,  cell wall galactomannoprotein Mp2/allergen  integral membrane protein  MFS allantoate transporter, putative  glycerophosphoryl diester phosphodiesterase  14-alpha sterol demethylase Cyp51A  conserved hypothetical protein  conserved hypothetical protein  conserved hypothetical protein  conserved hypothetical protein  MFS transporter, putative  hypothetical protein  GPI anchored protein, putative  conserved hypothetical protein  O-methyltransferase  glucokinase GlkA, putative  pathogenesis associated protein Cap20, putative  Nulp1-pending protein  acetamidase/formamidase family protein  extracellular serine-rich protein, putative  hypothetical protein  serum paraoxonase/arylesterase family protein  phosphatidate cytidylyltransferase, putative  integral membrane protein PTH11-like, putative  conserved hypothetical protein  amino acid permease (Gap1), putative  hypothetical protein  ferrooxidoreductase Fet3, putative  alkaline serine protease (PR1)/allergen  non-classical export protein Nce102, putative  conserved hypothetical protein  extracellular proline-serine rich protein  conserved hypothetical protein  C6 transcription factor, putative  protein phosphatase type 1 complex subunit  glycan biosynthesis protein (PigL), putative  DUF1445 domain protein  conserved hypothetical protein  hypothetical protein  GNAT family acetyltransferase, putative  conserved hypothetical protein  conserved hypothetical protein  glycine dehydrogenase  conserved hypothetical protein  class V chitinase, putative  67 kDa myosin-cross-reactive antigen family  sensor histidine kinase/response regulator,  C6 transcription factor, putative  bZIP transcription factor, putative  integral membrane protein  carboxypeptidase S1, putative  oligopeptide transporter, putative  hypothetical protein  heat shock transcription factor Hsf1, putative  flavin-binding monooxygenase, putative  cellobiose dehydrogenase  caffeine-induced death protein Cid2, putative  conserved hypothetical protein  endosomal SPRY domain protein, putative  NmrA-like family protein  MFS toxin efflux pump (AflT), putative  FAD dependent oxidoreductase superfamily  hypothetical protein  hypothetical protein  zinc-binding oxidoreductase, putative  MFS multidrug transporter, putative  Ankyrin and HET domain protein  GTP binding protein (Bud4), putative  conserved hypothetical protein  MYB DNA-binding domain protein  pheromone P-factor (Map2), putative  1,3-beta-glucanosyltransferase, putative  glycosyl transferase family 8 family, putative  conserved hypothetical protein  conserved hypothetical protein  protein phosphatase 2C, putative  conserved hypothetical protein  alpha-ketoglutarate-dependent taurine  Pfs, NACHT, and Ankyrin domain protein  nitrate transporter CrnA  HET domain protein  conserved hypothetical protein  Glutamate/Leucine/Phenylalanine/Valine  MFS alpha-glucoside transporter, putative  hypothetical protein  VPS9 domain protein, putative  2,3-diketo-5-methylthio-1-phosphopentane  SUN domain protein (Adg3), putative  conserved hypothetical protein  ABC multidrug transporter Mdr1  conserved hypothetical protein  MFS transporter, putative  ammonium transporter MeaA  hypothetical protein  SPX domain protein  conserved hypothetical protein  conserved hypothetical protein  extracellular dioxygenase, putative  hypothetical protein  conserved hypothetical protein  cytochrome P450 alkane hydroxylase, putative  pyridine nucleotide-disulphide oxidoreductase  conserved hypothetical protein  regulator of secondary metabolism LaeA  hypothetical protein  conserved hypothetical protein  purine-cytosine permease  zinc-containing alcohol dehydrogenase, putative  oxidoreductase, 2OG-Fe(II) oxygenase family,  conserved hypothetical protein  conserved hypothetical protein  OB-fold nucleic acid binding domain  integral membrane protein Pth11-like, putative  conserved hypothetical protein  conserved predicted protein  alpha/beta hydrolase family protein, putative  conserved hypothetical protein  conserved hypothetical protein  short-chain dehydrogenase, putative  esterase family protein  F-box domain protein  uridine permease Fui1, putative  sodium P-type ATPase, putative  MFS peptide transporter, putaitve  high affinity nitrate transporter NrtB  hypothetical protein  hypothetical protein  hypothetical protein  C2H2 finger domain protein, putative  glycosyl transferase, putative  NF-X1 finger and helicase domain protein,  conserved hypothetical protein  L-asparaginase  ammonium transporter (Mep2), putative  integral membrane protein  alpha-1,3-glucanase, putative  cell surface metalloreductase (FreA), putative  DUF821 domain protein  ornithine carbamoyltransferase  integral membrane protein  small oligopeptide transporter, OPT family  conserved hypothetical protein | 285.255  149.158  32.708  30.142  17.182  7.964  5.625  4.843  4.757  4.744  4.357  4.215  4.213  4.063  3.974  3.807  3.543  3.428  3.347  3.155  3.151  2.975  2.842  2.684  2.668  2.632  2.627  2.614  2.564  2.488  2.426  2.379  2.377  2.322  2.278  2.219  2.196  2.142  2.097  2.074  1.949  1.926  1.918  1.899  1.888  1.873  1.835  1.827  1.826  1.803  1.797  1.771  1.768  1.764  1.760  1.747  1.735  1.691  1.676  1.671  1.670  1.670  1.654  1.653  1.624  1.614  1.612  1.607  1.585  1.584  1.582  1.574  1.560  1.555  1.552  1.547  1.547  1.545  1.525  1.523  1.503  1.503  0.662  0.654  0.650  0.649  0.648  0.648  0.647  0.647  0.645  0.645  0.644  0.644  0.643  0.643  0.639  0.636  0.634  0.634  0.634  0.632  0.630  0.630  0.626  0.625  0.624  0.624  0.624  0.616  0.611  0.611  0.609  0.606  0.602  0.602  0.601  0.601  0.599  0.596  0.596  0.595  0.592  0.592  0.588  0.587  0.587  0.586  0.586  0.585  0.582  0.582  0.581  0.580  0.580  0.577  0.576  0.575  0.573  0.573  0.573  0.572  0.571  0.569  0.568  0.568  0.564  0.563  0.562  0.560  0.559  0.557  0.557  0.552  0.550  0.548  0.548  0.548  0.547  0.545  0.545  0.544  0.543  0.542  0.541  0.540  0.540  0.539  0.537  0.537  0.536  0.536  0.535  0.529  0.522  0.521  0.519  0.518  0.517  0.502  0.502  0.500  0.500  0.498  0.495  0.494  0.493  0.491  0.490  0.490  0.490  0.489  0.489  0.481  0.479  0.477  0.476  0.475  0.474  0.473  0.471  0.467  0.466  0.465  0.462  0.462  0.457  0.454  0.453  0.453  0.451  0.449  0.445  0.445  0.443  0.442  0.434  0.433  0.432  0.432  0.429  0.428  0.422  0.422  0.419  0.417  0.415  0.409  0.407  0.405  0.396  0.394  0.391  0.382  0.381  0.381  0.376  0.375  0.373  0.370  0.366  0.365  0.363  0.363  0.361  0.360  0.360  0.359  0.357  0.353  0.351  0.348  0.344  0.344  0.339  0.338  0.335  0.333  0.332  0.330  0.330  0.328  0.327  0.322  0.321  0.320  0.313  0.310  0.310  0.304  0.303  0.298  0.294  0.293  0.290  0.288  0.285  0.271  0.262  0.259  0.258  0.254  0.251  0.248  0.248  0.246  0.242  0.242  0.239  0.236  0.236  0.232  0.229  0.213  0.213  0.208  0.208  0.203  0.202  0.201  0.198  0.197  0.196  0.196  0.195  0.191  0.190  0.188  0.175  0.174  0.174  0.166  0.165  0.163  0.162  0.160  0.153  0.149  0.147  0.147  0.146  0.146  0.146  0.143  0.132  0.131  0.130  0.127  0.125  0.124  0.124  0.123  0.122  0.121  0.118  0.116  0.114  0.113  0.111  0.111  0.104  0.101  0.097  0.097  0.096  0.096  0.092  0.092  0.091  0.090  0.082  0.077  0.076  0.074  0.074  0.074  0.072  0.072  0.068  0.065  0.061  0.060  0.058  0.050  0.048  0.045  0.041  0.037  0.034  0.030  0.023  0.023  0.013  0.007 | 0.003  0.017  0.033  0.019  0.003  0.003  0.003  0.038  0.010  0.028  0.030  0.016  0.009  0.003  0.019  0.004  0.003  0.011  0.007  0.040  0.027  0.031  0.000  0.014  0.003  0.041  0.021  0.001  0.024  0.034  0.003  0.002  0.006  0.038  0.006  0.011  0.007  0.005  0.007  0.038  0.040  0.019  0.019  0.004  0.020  0.038  0.015  0.001  0.010  0.013  0.019  0.031  0.006  0.033  0.012  0.007  0.019  0.030  0.010  0.004  0.003  0.009  0.005  0.037  0.018  0.020  0.039  0.040  0.026  0.025  0.009  0.008  0.017  0.040  0.034  0.015  0.001  0.020  0.023  0.023  0.005  0.023  0.004  0.024  0.006  0.016  0.003  0.004  0.011  0.020  0.030  0.015  0.009  0.004  0.002  0.025  0.009  0.032  0.043  0.013  0.004  0.014  0.006  0.015  0.004  0.035  0.019  0.021  0.024  0.001  0.039  0.001  0.011  0.029  0.043  0.007  0.004  0.036  0.002  0.001  0.013  0.037  0.043  0.019  0.004  0.042  0.014  0.033  0.005  0.032  0.016  0.002  0.019  0.004  0.008  0.010  0.007  0.004  0.014  0.034  0.009  0.004  0.013  0.015  0.037  0.024  0.010  0.015  0.033  0.024  0.019  0.026  0.003  0.038  0.009  0.003  0.033  0.004  0.010  0.043  0.032  0.022  0.030  0.001  0.024  0.005  0.007  0.023  0.042  0.014  0.005  0.019  0.019  0.043  0.001  0.004  0.043  0.001  0.003  0.032  0.021  0.014  0.007  0.002  0.005  0.001  0.001  0.001  0.021  0.008  0.005  0.012  0.042  0.015  0.037  0.021  0.039  0.026  0.004  0.004  0.019  0.026  0.004  0.001  0.019  0.009  0.023  0.028  0.004  0.001  0.014  0.002  0.019  0.003  0.017  0.031  0.043  0.022  0.008  0.011  0.016  0.037  0.013  0.025  0.025  0.005  0.001  0.015  0.002  0.028  0.011  0.033  0.003  0.032  0.017  0.015  0.007  0.021  0.039  0.001  0.010  0.001  0.014  0.027  0.002  0.005  0.027  0.027  0.032  0.016  0.002  0.023  0.032  0.012  0.014  0.008  0.004  0.004  0.004  0.005  0.024  0.005  0.003  0.007  0.019  0.003  0.030  0.009  0.007  0.021  0.009  0.003  0.001  0.031  0.005  0.016  0.011  0.008  0.002  0.002  0.015  0.037  0.005  0.024  0.033  0.004  0.014  0.015  0.011  0.001  0.002  0.004  0.036  0.043  0.037  0.027  0.001  0.014  0.004  0.007  0.009  0.043  0.007  0.028  0.013  0.029  0.039  0.035  0.023  0.002  0.001  0.002  0.010  0.040  0.003  0.008  0.024  0.005  0.004  0.007  0.009  0.018  0.013  0.004  0.002  0.001  0.028  0.041  0.013  0.020  0.001  0.003  0.035  0.036  0.002  0.004  0.011  0.006  0.013  0.001  0.017  0.031  0.007  0.027  0.005  0.024  0.036  0.003  0.021  0.009  0.015  0.004  0.031  0.005  0.006  0.001  0.002  0.023  0.004  0.021  0.003  0.010  0.003  0.001  0.015  0.006  0.015  0.008  0.003  0.008  0.007  0.001  0.002  0.001 |
